# A Systematic Review and Correlational Meta-Analysis of Factors Associated With Resilience of Normally Aging, Community-Living Older Adults

**DOI:** 10.1093/geront/gnab110

**Published:** 2021-08-04

**Authors:** Sylwia Górska, Anusua Singh Roy, Lucy Whitehall, Linda Irvine Fitzpatrick, Nichola Duffy, Kirsty Forsyth

**Affiliations:** School of Health Sciences, Queen Margaret University, Musselburgh, UK; School of Health Sciences, Queen Margaret University, Musselburgh, UK; School of Health Sciences, Queen Margaret University, Musselburgh, UK; Edinburgh Health and Social Care Partnership, NHS Lothian, UK; Edinburgh Health and Social Care Partnership, NHS Lothian, UK; School of Health Sciences, Queen Margaret University, Musselburgh, UK

**Keywords:** Analysis—meta-analysis, Analysis—systematic review, Contextual factors, Measurement, Personal factors

## Abstract

**Background and Objectives:**

Global policy emphasizes the need to promote healthy aging through supporting inclusivity, safety, and functional independence. Research indicates that efforts to enhance resilience can contribute to meeting these objectives. We employed a meta-analytical approach to examine evidence on resilience in community-living older adults.

**Research Design and Methods:**

We searched electronic databases until January 13, 2020 for observational studies investigating factors associated with resilience in this population. Articles had to provide quantitative data based on standardized assessment and include samples where mean participants’ age and lower 95% confidence interval were more than 55 years. We included 49 studies reported in 43 articles and completed 38 independent meta-analyses, 27 for personal and 11 for contextual factors associated with resilience.

**Results:**

A range of personal and contextual factors were significantly associated with resilience, with effects sizes predominantly small to moderate (0.1 < *r* < 0.49). Factors reflecting psychological and physical well-being and access to/quality of social support were associated with higher resilience. Factors indicative of poorer psychological well-being and social challenges were associated with lower resilience. Longitudinal evidence was limited. The level of between-study heterogeneity was substantial to considerable. Where relevant analysis was possible, the identified publication bias was also considerable.

**Discussion and Implications:**

The quality of the available evidence, as well as issues related to measurement of resilience, indicates the need for further work relative to its conceptualization and assessment. The presented findings have important clinical implications, particularly within the context of the coronavirus disease 2019 impact on resilience in older adults.

## Background

People worldwide are living longer. By 2050, one in six people in the world will be older than 65 (16%), up from one in 11 in 2019 (9%; United Nations [UN], 2019). Aging presents both challenges and opportunities at individual and societal levels (Storey, 2018; World Health Organization [WHO], 2018). Consequently, governments internationally have been called to develop innovative policies and public services targeted specifically at older adults and aiming to support healthy lives and well-being by enhancing inclusivity, safety, and resilience within communities (Dugarova, 2017; Ziglio, 2017).

Traditionally, aging has been associated with frailty, vulnerability, and loss (Bartley et al., 2019). However, there is considerable variability in the aging process (WHO, 2015). People have an intrinsic capacity for positive adaptation throughout their life course (WHO, 2015) which, when supported by their environment, can be used to compensate for loss and changes associated with aging (Wallace et al., 2001). This capacity to positively adapt in response to adversity is called resilience (Lazarus, 1993; Ong et al., 2009). Research suggests that resilience supports the holistic view of healthy aging, predicting happiness, life satisfaction, and self-rated health (Fullen et al., 2018; Moore et al., 2015), and buffers against progression of disability (Manning et al., 2016). Therefore, efforts to boost resilience in older adults are of the utmost relevance, particularly in the context of the coronavirus disease 2019 (COVID-19) pandemic, as older adults are known to be disproportionately affected in terms of physical and mental health and well-being (UN, 2020).

In order to accurately assess resilience and develop effective interventions, clinicians must have at their disposal tools that accurately capture resilience (Cosco et al., 2016). The development of such tools reflects the way resilience is conceptualized (Bartley et al., 2019; Clark et al., 2019). Historically, resilience has been defined as a trait-like construct, consisting of personality characteristics and stable psychosocial factors that contribute to adaptive functioning (Block & Block, 1982; Rutter, 1985; Wagnild & Young, 1993). However, this has been challenged for overlooking time-varying and contextual aspects of resilient coping, as well as a failure to account for the malleability of human functioning or to consider how resilience can be promoted through therapeutic intervention (Bartley et al., 2019; Luthar et al., 2000). More recent theoretical perspectives conceptualize resilience as a dynamic adaptive capacity, built over time in response to adverse events experienced over the life course (Clark et al., 2018). This process-based theory positions resilience as an outcome of dynamic, complex interplay between multiple personal and contextual dimensions (Clark et al., 2018; Ong et al., 2009). Indeed, many studies (Bartley et al., 2019; Fullen et al., 2018; Li et al., 2015) have identified a range of personal (e.g., age, depressive symptoms, life satisfaction, and self-rated health) and contextual (e.g., education, income, and social support networks) factors which influence the resilience of older adults (see Supplementary Tables 7 and 8 for full lists of influential factors and related references).

Such a perspective recognizes resilience as a malleable factor that can be supported by targeted interventions (Bartley et al., 2019). Despite this, previously evaluated resilience interventions have tended to focus on enhancing protective factors within the individual (Lee et al., 2013). This is perhaps unsurprising given that much of the existing literature is focused on psychological resources (Bartley et al., 2019; Cosco et al., 2016; Windle et al., 2011). Moreover, while current research evidence recognizes a multisystem view of resilience (Bolton et al., 2016; Liu et al., 2017; MacLeod et al., 2016), there is neither consensus over its definition nor a “gold standard” for assessing resilience (Cosco et al., 2016; Windle et al., 2011). Existing definitions lack precision and fail to account for the multifaceted nature of resilience (Bartley et al., 2019). Resilience factors have been predominantly examined in isolation, overlooking their potentially synergistic and additive effects (Bartley et al., 2019). Consequently, dominant measures reflect trait-like conceptualization of resilience (Cosco et al., 2016; Windle et al., 2011), with a few, more recent tools attempting to capture its multidimensional nature (Martin, Distelberg et al., 2015) and none providing a comprehensive basis for measurement. Hence, the need to better understand multiple determinants of resilience and develop assessment tools that would more accurately reflect this knowledge. Such developments would allow health and social care professionals to more precisely distinguish older adults able to adapt after experiencing adversity and enable the development of targeted supports and interventions that address the individual and contextual factors for those who may struggle (Browne-Yung et al., 2017; Wallace et al., 2001), assisting global efforts to develop sustainable and equitable care systems for our older adults (WHO, 2017).

To support this, it appears timely to take stock of existing evidence. Previous reviews explored conceptual foundations of resilience in general populations (Dyer & McGuinness, 1996; Earvolino-Ramirez, 2007) and more specific contexts, for example, in the fields of aging (Cosco et al., 2015), youth mental health (Winders, 2014), or Aboriginal communities (Fleming & Ledogar, 2008). Systematic approaches were used to scrutinize psychometric rigor of resilience scales for general (Windle et al., 2011) and older adult (Cosco et al., 2016) populations. Bolton et al. (2016) offered a qualitative meta-synthesis of protective factors in older adults, while Hicks and Conner (2014) completed a concept analysis of resilient aging. A number of comprehensive reviews focused on resilience in older adults are also available (Fontes & Neri, 2015; MacLeod et al., 2016; Madsen et al., 2019). Lee et al. (2013) applied a meta-analytic methodology to identify risk and protective factors related to resilience across the life span. To date, no meta-analytic approach was applied to factors associated with resilience in community-living older adults. Such a review is needed to summarize the evidence as, given the contextual nature of resilience (Vanderbilt-Adriance & Shaw, 2008), it seems inappropriate to directly translate these general population-level findings to older adults.

In this systematic review and meta-analysis, we examined evidence from quantitative observational studies to identify factors associated with resilience in community-living older adults. We anticipate this knowledge to aid service providers in designing multidimensional interventions aimed at enhancing older adults’ resilience and achieve better personal outcomes, while remaining active, independent members of their communities; a flagship policy target internationally (WHO, 2017).

## Method

### Protocol and Registration

This systematic review and meta-analysis were conducted in accordance with Preferred Reporting Items for Systematic Reviews and Meta-Analyses guidelines (Supplementary Table 1; Knobloch et al., 2011). The review protocol was registered with PROSPERO: CRD42019162714.

### Search Strategy

We searched Abstracts in Social Gerontology, CINAHL, MEDLINE, ProQuest Central, PsycINFO, and Scopus for English language publications until January 13, 2020. The search string comprised (resilien* OR coping OR cope OR adapt* OR adjust* OR hardiness) AND (older adult* OR aging OR aging OR aged OR old age OR elderly) AND (community living OR community dwelling OR home OR independent living) AND (protective factor* OR risk factor* OR influencing factor* OR predictor* OR correlate* OR variable* OR demograph* OR resilien* scale). MeSH headings, free text searching, Boolean operators, and truncations were used to expand the literature search. No publication date restrictions were applied. The last searches were completed on January 13, 2020.

Records were downloaded into Reference Manager and screened against inclusion and exclusion criteria. Reference lists of relevant review articles identified through searches as well as articles meeting our predefined inclusion criteria were examined for additional publications.

### Inclusion and Exclusion Criteria

Older adults were the population of interest in this review. To allow for different conceptualizations of “old age” across different countries (WHO, 2002), we set a lower age limit at 55. We excluded studies where a mean age and lower 95% confidence interval (CI) were less than 55. Where articles included participants younger than the age of 55, lower 95% CIs were calculated using the mean age and the standard deviation (*SD*) of each sample, using the formula $\;\bar{x}-1.96\left(\sigma /\surd n\right)$, where $\;\bar{x}$ is the sample mean, σ is the *SD*, and *n* is the sample size (Lane, 2020). Three studies failed to report their samples’ age as a mean with the *SD*. King and Richardson (2016) reported the mean age and the age range of their participants. To ensure that this study met the inclusion criteria, the *SD* of the sample mean was estimated using the range rule for *SD* (σ ≈ (*b*−*a*)/4, where “*a*” is the minimum value and “*b*” is the maximum value; Ramírez & Cox, 2012). Calculating the *SD* then enabled the lower CI for the mean age to be estimated. Similar methods have been reported in previous meta-analyses (Jotheeswaran et al., 2016; Whitehall et al., 2021). Moore et al. (2015) only reported the mean age of their participants; however, they used the same data set as Jeste et al. (2013) which had a lower 95% CI of 76.55 years. Finally, Scelzo et al. (2018) only reported age ranges of their participants; consequently, the lower 95% CI of their samples’ age could not be calculated. Nevertheless, the decision was made to include their study in this meta-analysis as the reported sample characteristics suggest that the 95% CI for this study would have made it eligible for inclusion (age range: 51–101), and its exclusion may have caused theoretically important information to be lost.

Our focus was on normally aging seniors, with “normal aging” reflecting a biological norm (Canguilhem, 1991). An international review reported that approximately 62% of all people aged between 65 and 74 years and 81.5% of people of 85 years or older live with multiple conditions (Salive, 2013). Therefore, we defined normal aging as aging with a chronic disease (O’Rourke & Ceci, 2013). We excluded studies involving people with dementia as cognitive impairment in dementia deviates from the subtle age-related declines attributed to the process of “normal aging” (e.g., slower thinking and reduced attention; WHO, 2019). Consequently, the factors associated with the resilience of people with dementia may substantially differ from the general community-dwelling population of older adults (Christie, 2020). The focus of the current policy is to enable older adults to live within their communities for as long as possible (WHO, 2017). We therefore consider factors that shape resilience in community-living populations.

The review included observational studies providing cross-sectional or longitudinal data. Intervention studies were excluded as a pilot database search returned no interventions studies that provided the required data. Only data obtained with the use of standardized resilience measures were included. Based on conceptual underpinnings, these measures were classified as assessing either “trait resilience” or “resilience as coping process” (Supplementary Table 2).

### Procedure

**Figure 1.**
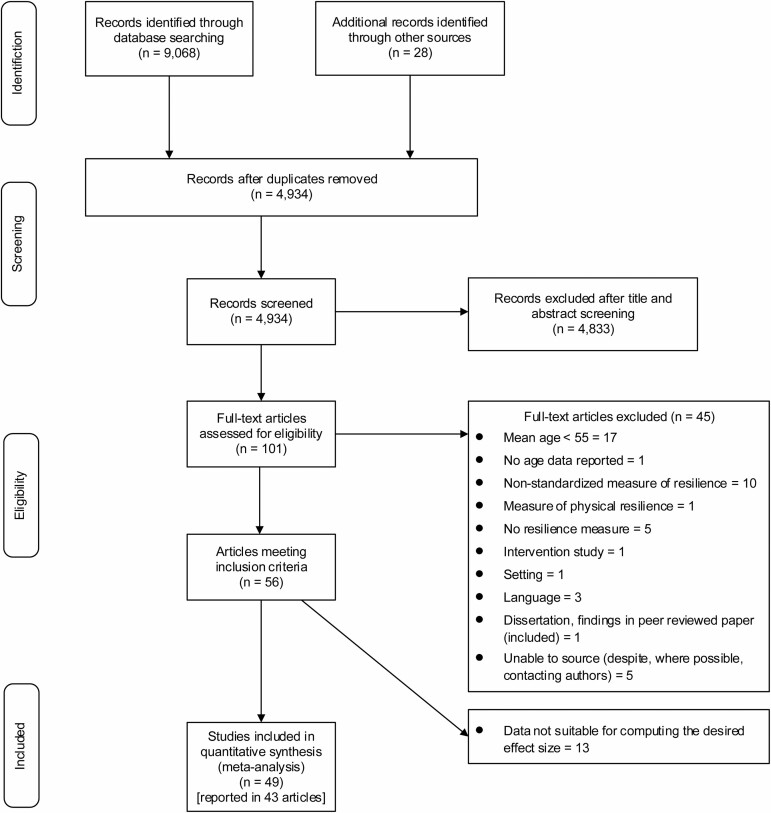
Study screening profile.

Figure 1 outlines the screening profile. Two reviewers (S. Górska and L. Whitehall) completed title, abstract, and full-text screening independently, using structured proforma. Any disagreements were referred to a third researcher (A. Singh Roy) for resolution. Study quality was assessed by two reviewers independently, using the National Heart, Lung and Blood Institute (National Institutes of Health [NIH]) Quality Assessment Tool for Observational Cohort and Cross-sectional Studies (NIH, 2020; Supplementary Table 3). Studies were categorized based on NIH (2020) quality rating into three categories: “good,” “fair,” or “poor.” A structured proforma was used for data extraction (Aromataris & Munn, 2020; Supplementary Table 4). Where multiple articles reported data from the same sample (Jeste et al., 2013; Martin, Palmer et al., 2015; Moore et al., 2015; Smith, 2009, 2012), appropriate effect size measures were included once only. If an article reported data for more than one independent sample (Martin, Palmer et al., 2015; Ong et al., 2006; Scelzo et al., 2018; Wagnild, 2003; You & Park, 2017), these were classed as separate studies.

### Statistical Analysis

Meta-analyses were undertaken to quantitatively synthesize data extracted from studies and consolidate information relating to the factors associated with resilience. A separate meta-analysis was conducted for each factor, using effect sizes based on correlation coefficients between the two continuous variables measuring resilience and the respective factor. Most studies directly reported a correlation coefficient (*r*). For others, reporting a standardized regression coefficient (β), the corresponding correlation coefficient was imputed using the formula: *r* = β + 0.05λ, where −0.50 ≤ β ≤ 0.50 and β is calculated from a single-equation linear regression model at the individual level; λ is an indicator variable where λ = 1 when β > 0 and λ = 0 when β < 0 (Peterson & Brown, 2005). Fully adjusted regression models were used in the imputation process, except for the study by Liddell and Ferreira (2019), where models were selected on the basis of the specific variables they adjusted for, and preference was given to the model that adjusted for a greater number of variables.

Correlation coefficients extracted for each study were converted to the Fisher’s *z* scale for its variance stabilization and normalization properties, where $z=\;0.5\;ln\left(\frac{1+r}{1-r}\right)$; $SE_{z}=\;\sqrt{\frac{1}{n-3}}$ (Borenstein et al., 2009). These transformed values were used to estimate the summary effect size and CI by fitting random-effects models, and the results were back-transferred to correlation coefficients. Estimated effect sizes (hereafter “effect sizes”) ≤0.09 were considered negligible, 0.10–0.29 small, 0.30–0.49 medium, and ≥0.50 large (Cohen, 1988). Visual representation of results, via forest plots, displayed the pooled effect size for each factor.

The presence of between-study variation was examined using the χ ^2^ test for heterogeneity that determines if the observed differences in results are due to random chance (Higgins et al., 2019). The amount of heterogeneity was quantified using the *I*^2^ statistic, which depicts the percentage of variation in estimated effects that is due to actual variation between studies rather than sampling error (Higgins et al., 2003). Leave-one-out sensitivity analysis was performed to further verify consistency and robustness of results obtained and consequently identify the sources of heterogeneity. Detection of possible publication bias via funnel plots was undertaken, wherein the standard error of estimates was plotted against the estimated effect sizes for each meta-analysis. For factors where at least 10 studies were included in the meta-analysis, funnel plot asymmetry was examined in order to identify the presence of bias (Higgins et al., 2019).

## Results

The search of online databases and other sources identified 9,096 publications. Following the screening procedure (Figure 1), 56 articles were identified as meeting inclusion criteria. Among these, 43 papers reported correlational data from 49 independent studies, completed across 10 countries: the United States (33), China (2) and Brazil, Germany, Italy, Norway, Sweden, Philippines, Singapore, and South Korea (1 each).

Of these, all but two studies (Manning et al., 2016; Silverman et al., 2015) were of cross-sectional design. The majority (24) were of “fair” quality indicating a moderate risk of bias, while 12 demonstrated “good” (low risk of bias) and seven “poor” (high risk of bias) quality. Where the risk of bias was identified, it was due to methods of sample selection, sample size and its justification, measurement standardization, and/or clarity regarding control for confounders. Supplementary Table 5 presents detailed characteristics of studies included in the meta-analysis; Supplementary Table 6 lists the excluded studies.

### Measures of Resilience

Eight standardized measures of resilience were used across the included studies (Supplementary Table 2). There were five measures of trait resilience, with the Resilience Scale (Wagnild & Young, 1993) being utilized most frequently. Six studies used measures of resilience as a coping process, with two each utilizing the Brief Resilience Scale (Smith et al., 2008), Resilience Appraisal Scale (Johnson et al., 2010), and Hardy–Gill Resilience Scale (Hardy et al., 2004).

Factors associated with resilience were categorized into personal and contextual. The complex nature of both resilience and influential factors can make it difficult to assign these factors into distinct categories (Hayman et al., 2017; Ungar, 2013; Vanderbilt-Adriance & Shaw, 2008). For example, loneliness can be conceptualized as an individual’s subjective feeling of psychological distress (personal) in response to perceived deficits in the number and quality of one’s social relationships (contextual) (Hawkley & Cacioppo, 2010; Matthews et al., 2021; Yanguas et al., 2018). To manage this complexity, for the purpose of this review, we applied criteria similar to those used by Hincks (2014) relative to factors associated with the concept of quality of life. Namely, we defined contextual factors as related to any objective or subjective indicator of the adversity (e.g., perceived stressfulness of the event) or a person’s physical, cultural, social, and economic environments (e.g., education, discrimination, and family/friend network size). In contrast, personal factors relate directly to the individual and reflect their values, beliefs, and feelings (e.g., life satisfaction and loneliness), their health and body functions (e.g., frailty and depressive symptoms), and their motor, process, and social interaction skills (e.g., cognitive functioning and social engagement).

### Studies Meeting Inclusion Criteria

Fifty-six articles met the inclusion criteria. These reported associations between resilience and 48 personal and 23 contextual factors. However, because some factors’ associations were reported by one study only, and 13 articles reported data not suitable for computing the required effect size, meta-analysis was infeasible for 21 personal and 12 contextual factors. All identified factors and, where relevant, reasons for exclusion from the meta-analysis are presented in Supplementary Tables 7 and 8.

### Studies Included in Meta-Analysis

**Figure 2.**
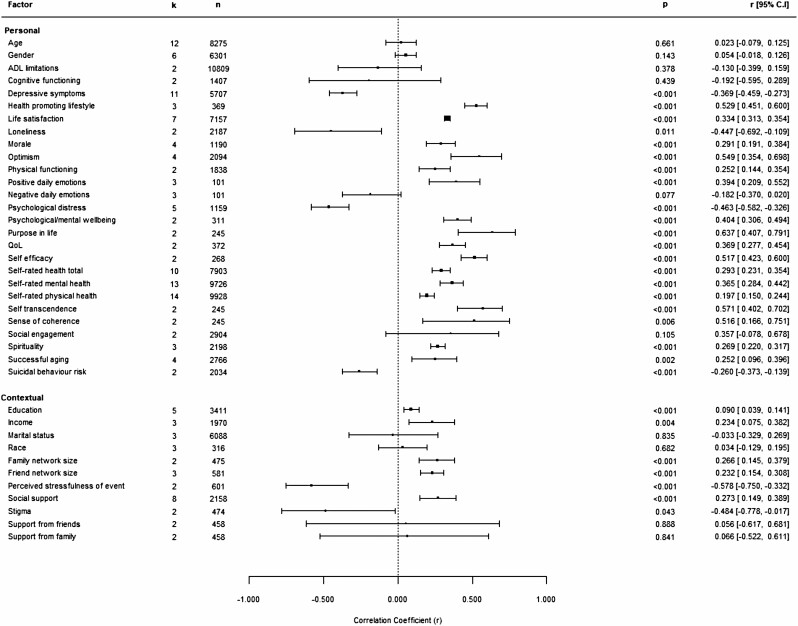
Forest plot showing estimated correlation coefficients between personal and contextual factors and resilience (combined measurement of resilience as trait and as a coping process). *Note:* Positive scores indicate that factors were related to higher resilience, and negative scores indicate that factors were related to lower resilience. ADL = activities of daily living.

We completed meta-analyses for the identified factors where the available data were supported by measurement reflecting either resilience as a trait or coping process. This resulted in 38 independent meta-analyses (27 personal and 11 contextual factors), based on sample sizes ranging from 101 to 10,809 participants and 2–14 studies. Figure 2 shows a forest plot of the pooled correlation coefficients across studies measuring the association between resilience and each factor. It also shows the number of studies and total sample size across which effect sizes were combined.

**Figure 3.**
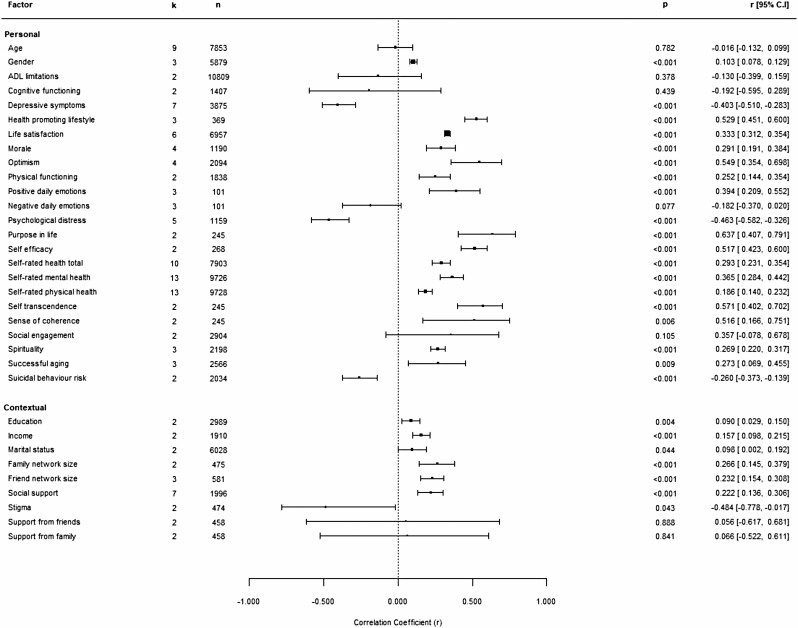
Forest plot showing estimated correlation coefficients between personal and contextual factors and trait resilience. *Note:* Positive scores indicate that factors were related to higher resilience, and negative scores indicate that factors were related to lower resilience. ADL = activities ofdaily living.

**Figure 4.**
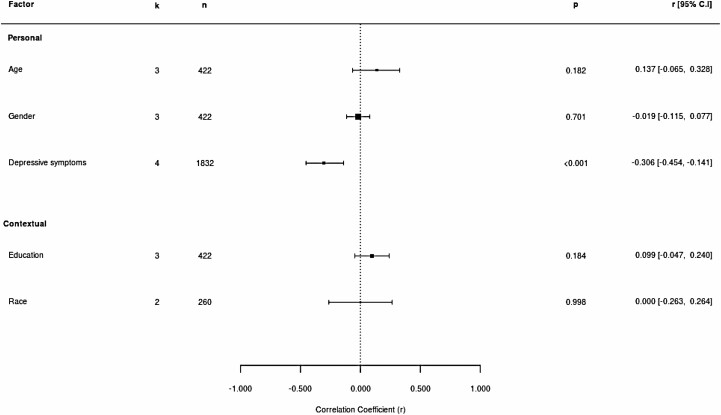
Forest plot showing estimated correlation coefficients between personal and contextual factors and resilience as a coping process. *Note:* Positive scores indicate that factors were related to higher resilience, and negative scores indicate that factors were related to lower resilience.

Where sufficient data were available, we completed a separate analysis based on the type of resilience measurement, resulting in 33 meta-analyses across personal and contextual factors for measurement of resilience as a trait and five meta-analyses across personal and contextual factors for resilience measured as a coping process. Figures 3 and 4 show forest plots illustrating these analyses.

### Resilience and Personal Factors

Statistically significant relationships (5% level of significance) were found between resilience and a number of personal factors. Effect sizes ranged from small to large, indicating poor to strong associations between resilience and personal factors.

#### Personal factors associated with higher resilience

Higher scores on measures of health-promoting lifestyle, optimism, purpose in life, self-efficacy, self-transcendence, and sense of coherence showed strong (≥0.50) positive associations with resilience, regardless of the conceptual basis behind the resilience measure used. Life satisfaction, morale, positive daily emotions, spirituality, successful aging, self-rated composite health, self-rated mental health, self-rated physical health, and physical functioning were also positively related to resilience, regardless of the approach to measurement. These associations were low to moderate (0.1 < *r* < 0.49). Psychological well-being and quality of life both showed positive, moderate associations with resilience. For both factors, due to the low number of studies underpinning the analysis, only a combined analytical approach was possible. Gender was the only sociodemographic factor weakly correlated with trait resilience, suggesting higher trait resilience for females. However, this relationship was not supported by analysis combining data across approaches to measurement or data based on measurement of resilience as a coping process only.

#### Personal factors associated with lower resilience

Depressive symptoms were moderately, negatively related to resilience regardless of the approach to measurement. Loneliness showed moderate negative associations with resilience in combined analysis as, due to a low number of studies, only this approach was possible. Psychological distress was moderately, negatively related to trait resilience. Also based on data reflecting resilience as a trait, the risk of suicidal behavior showed a weak, negative association with resilience.

### Resilience and Contextual Factors

A number of contextual factors were significantly associated with resilience (5% level of significance). Estimated effect sizes were predominantly small, indicating low strength of associations. Only one factor reached medium and one large effect size.

#### Contextual factors associated with higher resilience

Education, income, family/friend network size, and social support were all weakly correlated with resilience. The relationship between education and resilience became statistically nonsignificant when only data based on measurement of resilience as a coping process were considered. Marital status was weakly associated with trait resilience, but not when data across types of resilience measurement were analyzed together.

#### Contextual factors associated with lower resilience

Perceived stressfulness of event showed a strong, negative association with resilience. This was based on two studies, representing a different conceptual basis for measuring resilience. Experienced stigma was moderately, negatively related to trait resilience.

### Factors Not Significantly Associated With Resilience

Personal factors showing statistically nonsignificant associations with resilience, found across types of resilience measurement, include age, gender, activities of daily living limitations, cognitive functioning, negative daily emotions, and social engagement. Marital status, race, and support from family/friends were among contextual factors nonsignificantly associated with resilience when combined measurement was used in the analysis.

### Heterogeneity

Table 1 illustrates the measures assessing heterogeneity between studies for each factor—the χ ^2^ test statistic, *Q*, and its *p* value, and the *I*^2^ statistic and its CI.

**Table 1.** Between-Study Heterogeneity for Meta-Analysis Corresponding to Each Factor

Factor	*k*	*n*	*r* [95% CI]	*r*_p_	*Q*	*Q*_p_	*I* ^2^ [95% CI]
*Personal*							
Age	12	8,275	0.023 [−0.079, 0.125]	0.661	67.67	<0.001	83.74% [73.06%, 90.19%]
Gender	6	6,301	0.054 [−0.018, 0.126]	0.143	7.35	0.196	31.99% [0.00%, 72.50%]
ADL limitations	2	10,809	−0.13 [−0.399, 0.159]	0.378	4.87	0.027	79.47% [11.35%, 95.25%]
Cognitive functioning	2	1,407	−0.192 [−0.595, 0.289]	0.439	12.87	<0.001	92.23% [73.43%, 97.73%]
Depressive symptoms	11	5,707	−0.369 [−0.459, −0.273]	<0.001	193.51	<0.001	94.83% [92.45%, 96.46%]
Health-promoting lifestyle	3	369	0.529 [0.451, 0.6]	<0.001	0.00	0.998	0.00% [0.00%, 0.00%]
Life satisfaction	7	7,157	0.334 [0.313, 0.354]	<0.001	7.06	0.315	15.06% [0.00%, 58.81%]
Loneliness	2	2,187	−0.447 [−0.692, −0.109]	0.011	21.19	<0.001	95.28% [86.01%, 98.41%]
Morale	4	1,190	0.291 [0.191, 0.384]	<0.001	5.93	0.115	49.45% [0.00%, 83.27%]
Optimism	4	2,094	0.549 [0.354, 0.698]	<0.001	88.84	<0.001	96.62% [93.86%, 98.14%]
Physical functioning	2	1,838	0.252 [0.144, 0.354]	<0.001	2.94	0.087	65.96% [0.00%, 92.28%]
Positive daily emotions	3	101	0.394 [0.209, 0.552]	<0.001	0.05	0.976	0.00% [0.00%, 0.00%]
Negative daily emotions	3	101	−0.182 [−0.37, 0.02]	0.077	1.35	0.509	0.00% [0.00%, 84.61%]
Psychological distress	5	1,159	−0.463 [−0.582, −0.326]	<0.001	8.93	0.063	55.19% [0.00%, 83.45%]
Psychological well-being	2	311	0.404 [0.306, 0.494]	<0.001	0.93	0.334	0.00%
Purpose in life	2	245	0.637 [0.407, 0.791]	<0.001	6.42	0.011	84.43% [36.2%, 96.2%]
Quality of life	2	372	0.369 [0.277, 0.454]	<0.001	0.300	0.584	0.00%
Self-efficacy	2	268	0.517 [0.423, 0.6]	<0.001	0.300	0.586	0.00%
Self-rated general health	10	7,903	0.293 [0.231, 0.354]	<0.001	46.59	<0.001	80.68% [65.40%, 89.21%]
Self-rated mental health	13	9,726	0.365 [0.284, 0.442]	<0.001	137.97	<0.001	91.30% [86.96%, 94.20%]
Self-rated physical health	14	9,928	0.197 [0.150, 0.244]	<0.001	30.19	0.004	56.95% [21.86%, 76.28%]
Self-transcendence	2	245	0.571 [0.402, 0.702]	<0.001	3.08	0.079	67.57% [0.00%, 92.67%]
Sense of coherence	2	245	0.516 [0.166, 0.751]	0.006	10.12	0.001	90.12% [63.86%, 97.3%]
Social engagement	2	2,904	0.357 [−0.078, 0.678]	0.105	152.62	<0.001	99.34% [98.81%, 99.64%]
Spirituality	3	2,198	0.269 [0.22, 0.317]	<0.001	1.61	0.448	0.00% [0.00%, 87.04%]
Successful aging	4	2,766	0.252 [0.096, 0.396]	0.002	76.37	<0.001	96.07% [92.65%, 97.90%]
Risk of suicidal behavior	2	2,034	−0.26 [−0.373, −0.139]	<0.001	8.15	0.004	87.73% [52.48%, 96.83%]
*Contextual*							
Education	5	3,411	0.09 [0.039,0.141]	<0.001	7.02	0.135	43.00% [0.00%, 79.05%]
Income	3	1,970	0.234 [0.075,0.382]	0.004	7.43	0.024	73.08% [9.56%, 91.99%]
Marital status	3	6,088	−0.033 [−0.329,0.269]	0.835	14.84	<0.001	86.53% [61.22%, 95.32%]
Race	3	316	0.034 [−0.129,0.195]	0.682	3.46	0.178	42.15% [0%, 82.49%]
Family network size	2	475	0.266 [0.145,0.379]	<0.001	1.92	0.166	47.78%
Friend network size	3	581	0.232 [0.154,0.308]	<0.001	0.78	0.678	0.00% [0.00%, 73.24%]
Perceived stressfulness of the event	2	601	−0.578 [−0.75, −0.332]	<0.001	5.01	0.025	80.05% [14.21%, 95.36%]
Social support	8	2,158	0.273 [0.149,0.389]	<0.001	42.74	<0.001	83.62% [69.28%, 91.27%]
Stigma	2	474	−0.484 [−0.778, −0.017]	0.043	28.29	<0.001	96.47% [90.35%, 98.71%]
Support from friends	2	458	0.056 [−0.617,0.681]	0.888	60.34	<0.001	98.34% [96.29%, 99.26%]
Support from family	2	458	0.066 [−0.522,0.611]	0.841	41.68	<0.001	97.60% [94.1%, 99.02%]

*Note:* CI = confidence interval; ADL = activities of daily living.

Because most factors include only a small number of studies and/or limited sample size, a more stringent threshold for statistical significance, *Q*_p_ < 0.10, was considered for the χ ^2^ test of heterogeneity in order to overcome its issue of low power (Higgins et al., 2019). The test yielded statistically significant variability between studies for the majority of factors, with corresponding *I*^2^ values quantifying this heterogeneity as substantial to considerable (Higgins et al., 2019). In cases where *Q*_p_ is ≥0.10, CIs for *I*^2^ are usually wide, with *I*^2^ = 0 in some instances.

The leave-one-out sensitivity analysis (Supplementary Table 9) identified a number of studies as influential and potential sources of heterogeneity, for example, Liddell and Ferreira (2019) for factors life satisfaction, self-rated health total, and gender; Li et al. (2015) for social support and gender; Lu et al. (2017) for optimism; and Bartley et al. (2019) for marital status and income. Omission of these studies affected the meta-analyses’ results in terms of the heterogeneity statistics, level of significance, and the estimated effect size. For factors marital status and gender, upon exclusion of the works of Bartley et al. (2019) and Li et al. (2015), respectively, effect sizes that were initially nonsignificant changed to small but significant. For income, optimism, and social support, effect sizes remained significant but slightly decreased in magnitude, whereas for self-rated health total and life satisfaction, they remained significant but slightly increased in magnitude when influential studies were omitted. More substantial changes were observed for between-study heterogeneity, wherein the *I*^2^ statistic considerably reduced in most cases on removal of these influential studies. Potential sources of heterogeneity linked to the studies identified as influential include (a) methodology applied to the computation of the effect size; (b) variations in conceptual basis behind the resilience measures used; (c) use of nonstandardized tools in measurement of continuous variables associated with resilience; (d) use of diverse coding for categorical variables associated with resilience; (e) participants’ characteristics, including cultural diversity between analyzed samples, focus on older adults living with a specific health condition or those living in postdisaster communities.

### Publication Bias

Funnel plots offering visualization of the bias analysis for factors with at least 10 studies are presented in Supplementary Figure 10A–E. Funnel plot asymmetry was substantially noted for factors self-rated health (total, physical, and mental), wherein smaller studies without statistically significant effects were likely unreported, causing gaps in the bottom corners of the plots. Possibility of bias was also detected for factors age and depressive symptoms, where several studies were outside the 95% confidence region based on a random-effects meta-analysis.

## Discussion

To the best of our knowledge, this is the first systematic review with meta-analyses of factors associated with resilience in community-living older adults. It is also the first such review including measures of resilience as a trait and coping process and to explicitly consider both personal and contextual factors associated with resilience. The majority of the included studies were cross-sectional, with substantial to considerable between-study heterogeneity. Most studies demonstrated a moderate to high risk of bias. From a broad range of factors identified as being related to resilience, about 50% were supported by evidence sufficient to facilitate meta-analysis. Where meta-analysis was possible, a number of personal and contextual factors were significantly related to resilience, with most showing weak to moderate and a few reaching strong associations. Where strong associations were found, CIs were typically wide. Only 13 of 38 meta-analyses were supported by data from more than three studies.

Among sociodemographic factors, age and race were unrelated to resilience. Gender, education, income, and marital status showed weak but inconsistent associations, depending on the type of resilience measurement. Our observations relative to relationships between sociodemographic factors and resilience resonate with the previous meta-analytical review, which highlighted the inconsistency of findings and relatively low effect of these factors on resilience when compared with the effect of other psychosocial influences (Lee et al., 2013).

In this meta-analysis, factors indicative of physical and psychological well-being were generally associated with higher resilience, as were those reflecting access to and quality of social support. The majority of these relationships were weak to moderate, with only a few personal factors demonstrating strong associations, including health-promoting lifestyle, optimism, purpose in life, self-efficacy, self-transcendence, and sense of coherence. This is consistent with findings of systematic reviews which sought to synthesize available data about older adults’ protective factors (Bolton et al., 2016; Earvolino-Ramirez, 2007; Resnick, 2011), as well as with other meta-analyses which investigated factors associated with resilience in different populations (Lee et al., 2013 [adults]; Yule et al., 2019 [children exposed to violence]).

We found that a number of personal factors (loneliness, depression, and psychological distress) were moderately associated with lower resilience. Additionally, two contextual factors (perceived stressfulness of the event and experience of stigma) showed similar patterns of association. Loneliness, depression, and psychological distress have previously been reported as being associated with lower resilience in older adults (Clark et al., 2018; Mlinac et al., 2011), as well as in other populations (Chai et al., 2019 [left-behind children]; Iacob et al., 2020 [familial caregivers]; Lee et al., 2013 [adults]). Higher perceived stressfulness of the event has also previously been identified as being associated with lower resilience (Hye Kyung et al., 2017 [nurses]; Lee et al., 2013 [adults]). However, our finding of experience of stigma being associated with lower resilience is relatively novel, although Hayman et al. (2017) suggest that a stigma of aging may negatively affect resilience.

Due to limited longitudinal data, we were unable to consider the role of the identified factors as predictors of resilience. Moreover, we recognize that the effect of relationships between resilience and some sociodemographic and psychosocial factors may vary depending on the approach to the measurement of resilience, as we found that the relationships between resilience and gender, marital status, and education differed based on the approach to measurement. This supports the notion that resilience results from complex associations across many domains, which may covary in different combinations to influence individual results (Bartley et al., 2019; Dahlberg, 2015; Southwick et al., 2014). It may also reflect a theory that health outcomes, including resilience, are influenced by many factors operating on many levels, and that this impact may vary over time and context (Hayman et al., 2017; Orford, 2008).

The importance of a range of personal and contextual factors relative to resilience has been identified in a previous meta-analysis focused on resilience across the life span (Lee et al., 2013). Our review identified a number of additional factors, for example, spirituality, purpose in life, self-rated physical and mental health, which were not identified by Lee et al. (2013). But, for some factors previously recognized as important, for example, self-esteem, negative affect, or anxiety (Lee et al., 2013), due to insufficient data, we were unable to complete the meta-analysis. This too aligns with the notion of the contextual nature of resilience (Vanderbilt-Adriance & Shaw, 2008), indicating that factors associated with resilience may change over the life span, supporting the need for a better understanding of its contextual determinants (Hayman et al., 2017). Consideration of our findings in the context of previous qualitative and comprehensive reviews highlights that a number of potentially important factors, for example, previous experience of hardship (Bolton et al., 2016; Hicks & Conner, 2014), altruism (Bolton et al., 2016), or cultural dimensions (Fleming & Ledogar, 2008), have not been quantitatively evaluated in older adults or, as shown in this review, there is insufficient quantitative data to support meta-analysis. This indicates that associations examined in quantitative studies to date, and certainly those captured in this meta-analysis, are unlikely to reflect all factors that are critical to understanding and supporting development of interventions aiming to promote resilience in older adults.

Studies included in our review employed numerous standardized measures of resilience, with the Resilience Scale (Wagnild & Young, 1993) being utilized most frequently. Previous reviews considered the measurement of resilience in adult (Windle et al., 2011) and older adult (Cosco et al., 2016) populations. Windle et al. (2011) highlight that despite wide recognition of resilience as being associated with personal and contextual factors, the vast majority of resilience tools capture only its individual domains. The same was acknowledged by Cosco et al. (2016) relative to tools used to assess resilience in older adults. Windle et al. (2011) recommend that, to facilitate development of effective interventions, resilience measures should reflect a multilevel perspective that spans personal and contextual determinants. However, although new tools, reportedly meeting this criterion (Martin, Distelberg et al., 2015), have been developed in the context of community-dwelling older adults, they neither capture all important aspects of resilience nor have established properties of validity and reliability, and as we reflect, are not widely used in research.

Due to scarcity of evidence, we took a decision to statistically analyze all factors for which data were available from two or more studies. Consequently, the number of studies included in each meta-analysis is generally small, with 77% including fewer than five studies. This approach allowed consideration of a wider range of factors than would be possible if we applied more stringent selection criteria. However, it affected the robustness of the average population effect size and average sampling error calculated. Lack of a substantial number of studies also affects the estimation of between-study variance because it causes the χ ^2^ test to have low power and uncertainty in the value of *I*^2^, that is, wider CIs. Moreover, inconsistent reporting of demographic data across included studies prevented meta-regression or subgroup analyses, meaning additional potential sources of heterogeneity could not be considered. For some studies, we had to impute the effect size, which further affected the accuracy of the analysis. Additionally, due to the nature of underlying data, we examined factors separately and could not account for likely intercorrelations. Finally, the completed publication bias analysis indicated the possibility of reporting biases that are likely to result in overestimation of effect estimates. In this context, publication of high-quality research on resilience in older adults, inclusive of negative findings, should be encouraged to facilitate a more accurate evaluation of evidence.

Our findings highlight some limitations relative to the lack of consistency in defining, conceptualizing, and measuring resilience in older adults, that is, we identified that the relationship between influential factors and resilience may vary depending on how resilience is measured. Although current conceptualizations emphasize the multidimensionality and dynamic nature of resilience (Liu et al., 2017), the prevailing approaches to the study of resilience fail to account for these characteristics (Bartley et al., 2019). Consequently, most established measures do not capture all relevant factors (Cosco et al., 2016; Windle et al., 2011) and none can serve as a “gold standard” for resilience assessment (MacLeod et al., 2016; Windle et al., 2011). This is important, as inaccurate measurement may provide misleading information, affecting the accuracy of research and clinical recommendations (Cosco et al., 2016; Hayman et al., 2017). Therefore, broadening the perspective to include a range of personal and contextual factors, conceptualized from different levels and reflecting both protective and risk factors, is likely to provide a greater understanding and basis for measurement of resilience. We concur with Bartley et al. (2019) that incorporating additional dimensions, reflecting health and lifestyle as well as a broader range of psychological and contextual factors, will be key to improving the understanding, assessment, and design of interventions to promote resilience in older adults. Such comprehensive consideration of resilience may also contribute to models of healthy aging as, through the addition of adversity and resilience to the healthy aging model, the concept becomes more appropriate for the aging population who are likely to experience a range of adversities (Cosco et al., 2017). A greater understanding of the influence of contextual factors on resilience may also support the design of environments and health systems that support healthy aging, through identifying social and community factors that support an individual’s ability to adapt well in the face of age-related adversities (Earvolino-Ramirez, 2007; Hayman et al., 2017; Hicks & Conner, 2014; Wong, 2018).

Improvements in this area will be of particular importance in the aftermath of the COVID-19 pandemic, which disproportionately affects older adults’ ability to navigate through and adapt to age-related and societal challenges we all experience as a result (Harkins, 2020; UN, 2020). It has been reported that, during the pandemic, resilience has moderated the relationship between stress and mental health outcomes (Havnen et al., 2020). However, preliminary research (Mental Health Foundation, 2020; Wister & Speechley, 2020) has also found that the pandemic has caused an increase in vulnerability factors (e.g., poor health, decreased social support and social engagement, reduced access to community services, increased social isolation and loneliness, worsening psychological and economic resources, and harmful coping strategies), which may have a detrimental influence on individuals’ resilience. At the same time, many protective factors (e.g., social engagement, contact with friends and family, income, and physical activity) have been negatively affected by the pandemic, particularly for older adults (Mental Health Foundation, 2020; Wister & Speechley, 2020). Consequently, innovative ways to bolster older adults’ resilience are needed (Fuller & Huseth-Zosel, 2021; Wister & Speechley, 2020), especially as it is recognized that the impact of the COVID-19 pandemic is likely to be long-lasting (The British Academy, 2021). Supporting the protective factors and identifying and addressing the vulnerability factors of older adults will be crucial as they face the continuing consequences of the pandemic (Wister & Speechley, 2020).

## Conclusions

This review highlighted limitations in prevailing ways of conceptualizing and assessing resilience, which may impede how services support older adults. Our findings support the need for conceptualization and measurement of resilience that would incorporate a broader range of personal and contextual dimensions, considered at different levels, and reflecting health and lifestyle as well as psychological and contextual factors. Additionally, there is a need for longitudinal research to reflect these changes, inform development of multidimensional interventions to promote resilience, and support identification of older adults who may benefit from a timely provision of preventative measures.

## Funding

This work was jointly supported by the National Health Service (NHS) Lothian and Queen Margaret University Edinburgh.

## Conflict of Interest

None reported.

## Supplementary Material

gnab110_suppl_Supplementary_Materials
