# Supplementary material for: A Systematic Review and Correlational Meta-Analysis of Factors Associated With Resilience of Normally Aging, Community-Living Older Adults
Source: Gerontologist. 2021 Aug 4;62(9):e520–33. doi: 10.1093/geront/gnab110 (PMC9579466; doi:10.1093/geront/gnab110)
Supplement: gnab110_suppl_Supplementary_Materials [file gnab110_suppl_supplementary_materials.docx]

**Supplementary Table 1**. PRISMA checklist


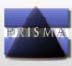
**PRISMA 2009 Checklist**

| **Section/topic** | **#** | **Checklist item** | **Reported in the section titled** |
| --- | --- | --- | --- |
| **TITLE** | | |  |
| Title | 1 | Identify the report as a systematic review, meta-analysis, or both. | Title |
| **ABSTRACT** | | |  |
| Structured summary | 2 | Provide a structured summary including, as applicable: background; objectives; data sources; study eligibility criteria, participants, and interventions; study appraisal and synthesis methods; results; limitations; conclusions and implications of key findings; systematic review registration number. | Abstract |
| **INTRODUCTION** | | |  |
| Rationale | 3 | Describe the rationale for the review in the context of what is already known. | Background |
| Objectives | 4 | Provide an explicit statement of questions being addressed with reference to participants, interventions, comparisons, outcomes, and study design (PICOS). | Background |
| **METHODS** | | |  |
| Protocol and registration | 5 | Indicate if a review protocol exists, if and where it can be accessed (e.g., Web address), and, if available, provide registration information including registration number. | Protocol and Registration |
| Eligibility criteria | 6 | Specify study characteristics (e.g., PICOS, length of follow-up) and report characteristics (e.g., years considered, language, publication status) used as criteria for eligibility, giving rationale. | Inclusion and Exclusion Criteria |
| Information sources | 7 | Describe all information sources (e.g., databases with dates of coverage, contact with study authors to identify additional studies) in the search and date last searched. | Search strategy |
| Search | 8 | Present full electronic search strategy for at least one database, including any limits used, such that it could be repeated. | Search strategy |
| Study selection | 9 | State the process for selecting studies (i.e., screening, eligibility, included in systematic review, and, if applicable, included in the meta-analysis). | Search strategy  Procedure |
| Data collection process | 10 | Describe method of data extraction from reports (e.g., piloted forms, independently, in duplicate) and any processes for obtaining and confirming data from investigators. | Procedure |
| Data items | 11 | List and define all variables for which data were sought (e.g., PICOS, funding sources) and any assumptions and simplifications made. | Statistical analysis  Supplementary Table 4 |
| Risk of bias in individual studies | 12 | Describe methods used for assessing risk of bias of individual studies (including specification of whether this was done at the study or outcome level), and how this information is to be used in any data synthesis. | Procedure  Supplementary Table 3 |
| Summary measures | 13 | State the principal summary measures (e.g., risk ratio, difference in means). | Statistical analysis |
| Synthesis of results | 14 | Describe the methods of handling data and combining results of studies, if done, including measures of consistency (e.g., I^2^) for each meta-analysis. | Statistical analysis |
| Risk of bias across studies | 15 | Specify any assessment of risk of bias that may affect the cumulative evidence (e.g., publication bias, selective reporting within studies). | Statistical analysis |
| Additional analyses | 16 | Describe methods of additional analyses (e.g., sensitivity or subgroup analyses, meta-regression), if done, indicating which were pre-specified. | Statistical analysis |
| **RESULTS** | | |  |
| Study selection | 17 | Give numbers of studies screened, assessed for eligibility, and included in the review, with reasons for exclusions at each stage, ideally with a flow diagram. | Results  Figure 1 |
| Study characteristics | 18 | For each study, present characteristics for which data were extracted (e.g., study size, PICOS, follow-up period) and provide the citations. | Results  Measures of resilience  Supplementary Table 5 |
| Risk of bias within studies | 19 | Present data on risk of bias of each study and, if available, any outcome level assessment (see item 12). | Supplementary Table 5 |
| Results of individual studies | 20 | For all outcomes considered (benefits or harms), present, for each study: (a) simple summary data for each intervention group (b) effect estimates and confidence intervals, ideally with a forest plot. | Results  Supplementary Table 7-8  Figures 2-4 |
| Synthesis of results | 21 | Present results of each meta-analysis done, including confidence intervals and measures of consistency. | Results  Figures 2-4 |
| Risk of bias across studies | 22 | Present results of any assessment of risk of bias across studies (see Item 15). | Heterogeneity  Table 1  Publication bias  Supplementary material 9 & 10a-e |
| Additional analysis | 23 | Give results of additional analyses, if done (e.g., sensitivity or subgroup analyses, meta-regression [see Item 16]). | Heterogeneity  Supplementary Table 9 |
| **DISCUSSION** | | |  |
| Summary of evidence | 24 | Summarize the main findings including the strength of evidence for each main outcome; consider their relevance to key groups (e.g., healthcare providers, users, and policy makers). | Discussion |
| Limitations | 25 | Discuss limitations at study and outcome level (e.g., risk of bias), and at review-level (e.g., incomplete retrieval of identified research, reporting bias). | Discussion |
| Conclusions | 26 | Provide a general interpretation of the results in the context of other evidence, and implications for future research. | Discussion |
| **FUNDING** | | |  |
| Funding | 27 | Describe sources of funding for the systematic review and other support (e.g., supply of data); role of funders for the systematic review. | Funding |

*From:*  Moher D, Liberati A, Tetzlaff J, Altman DG, The PRISMA Group (2009). Preferred Reporting Items for Systematic Reviews and Meta-Analyses: The PRISMA Statement. PLoS Med 6(7): e1000097. doi:10.1371/journal.pmed1000097

**Supplementary Table 2**. Resilience measures used in the included studies

| **Measurement tool** | **Type of resilience** | **Characteristics / conceptual foundations** | **Used in:** |
| --- | --- | --- | --- |
| The Resilience Scale (Wagnild & Young, 1993) | Trait Resilience | Captures personality characteristics of resilience (Resnick & Inguito, 2011),  25 items reflecting the five characteristics of resilience including:   - self-reliance or believing in oneself, - meaning, and the realization that life has purpose and meaning, - equanimity or acceptance of events that happen through life, - perseverance, which reflects persistence despite adversity, - existential aloneness or the realization that each person is unique and some experiences must be faced alone   Items fit into two factors: Personal Competence and Acceptance of Self and Life.  Scale from disagree (1) to agree (7).  Interpretation of scores:   - >145 indicate moderately high to high resilience - 126 – 145 moderately low to moderate resilience - ˂125 low resilience   Measure of resilience most frequently used with older adults (Resnick & Inguito, 2011).  Evidence supporting psychometric properties for older adults available in: Resnick & Inguito, 2011; Wagnild, 2003; Wagnild & Young, 1993. | 19 independent studies:  Coutto et al. 2011  Fraitag & Schmidt, 2016  King & Richardson, 2016  Lee et al. 2008  Manning et al. 2016  McClain et al. 2018  Moe et al. 2013  Nygren et al. 2005  Polson et al. 2018  Torma et al. 2013  Wagnild, 2003 (reporting 5 independent studies)  Wagnild & Torma, 2013  Wagnild & Young, 1993  Wells, 2009  Wells, 2010 |
| Connor Davidson Resilience Scale (Connor & Davidson, 2008) | Trait resilience | Captures personality aspects of resilience (Resnick & Inguito, 2011).  Main scale includes 25 items, all of which carry a 5-point range of responses, as follows: not true at all (0), rarely true (1), sometimes true (2), often true (3), and true nearly all of the time (4). The scale is rated based on how the subject has felt over the past month. The total score ranges from 0–100, with higher scores reflecting greater resilience (Connor & Davidson, 2003).  Three authorized versions: 25 item CD-RISC; 10 item CD-RISC-10; 2 item CD-RISC-2.  Other versions available but unauthorized due to substantial modifications (Davidson, 2020).  Psychometric properties established for various populations (Davidson, 2020). | 17 independent studies:  Jeste et al. 2013  Jeste et al. 2019  Kuwert et al. 2014  Lamond et al. 2008  Lim et al. 2015  Lu et al. 2017  Martin et al. 2015b  McKibbin et al. 2016  Montross et al. 2006  Moore et al. 2015  Scelzo et al. 2013  Schure et al. 2013  Silverman et al. 2015  Smith, 2009  Smith, 2012  Vahia et al. 2011  You & Park, 2007 |
| Dispositional Resilience Scale (Bartone et al., 1989) | Trait resilience | The theoretical background to the development of this scale is derived from the hardiness literature, and in a number of applications it is referred to as a measure of hardiness. As a personality style, it might assist in a resilient response from the individual level, however it is generally regarded as a fixed trait and does not fit well with the notion of resilience as a dynamic process. 3 dimensions, 45 items.  Psychometric properties reported: Bartone et al., 1989; Bartone, 1991; Bartone, 1995; Bartone, 2007. | 4 independent studies:  Ong et al. 2006 (two independent studies)  Rossi et al. 2007  Wallace et al. 2001 |
| Ego Resiliency Scale (Block & Kremen, 1996) | Trait resilience | Ego-resilience conceptualized as an aspect of personality which in turn served as a “structure for managing emotion” (Block, 2002).  Personality as an adaptive system for taking in and organizing information and maintaining non-disruptive levels of anxiety while responding to inner and outer demands. Block’s construct refers to a “personality trait” (Prince-Embury, 2013).  Fourteen items, scored 1-4 (does not apply at all – applies very strongly) (Block & Kremen, 1996). Scores from 0-56, with higher score representing higher resilience.  Psychometric properties reported by Block & Kremen (1996). | 2 independent studies:  Baldwin et al. 2011  Ong et al. 2006 |
| The Pearlin Mastery Scale (Pearlin & Schooler 1978) | Trait resilience | The Pearlin Mastery Scale (PM) measures an individual’s level of mastery, which is a psychological resource that has been defined as “the extent to which one regards one’s life-chances as being under one’s own control in contrast to being fatalistically ruled” (Pearlin & Schooler, 1978, p.5). Mastery, as an aspect of psychological coping, used as an indicator of resilience. The 7-item scale comprises five negatively worded items and two positively worded items, presented with the following response options: (1) Strongly Disagree (2) Disagree (3) Agree (4) Strongly Agree. The negatively worded items require reverse coding prior to scoring, resulting in a score range of 7 to 28, with higher scores indicating greater levels of mastery. | 1 study:  Liddell & Ferreira, 2019 |
| Brief Resilience Scale (Smith et al., 2008) | Resilience as coping process | Brief resilience scale was designed to determine whether it is possible to reliably assess resilience as a process of bouncing back from stress (Smith et al., 2008). It consists of 6 items, scored 1-5 (from ‘strongly agree’ to ‘strongly disagree’) but 3 positive, three negative statements implying reverse scoring for half of items.  Psychometric properties reported by Smith et al. (2008). | 2 independent studies:  Bartley et al. 2019  Fullen et al. 2018 |
| Hardy-Gill Resilience Scale  (Hardy, Concato & Gill, 2004) | Resilience as coping process | Hardy-Gill Resilience Scale assesses resilience as a coping process in response to a specific life event (Resnick & Inguito, 2011). Tool developed based on the construct of resilience as described by Rowe et al. (1997). Six items, scores ranging from 0 (low) to 18 (high).  Psychometric properties reported Hardy, Concato & Gill (2004). | 2 independent studies:  Hardy et al. 2004  Mehta et al. 2018 |
| Resilience Appraisal Scale (Johnson, Gooding, Wood & Tarrier, 2010) | Resilience as coping process | Rooted in the Schematic Appraisals Model of Suicide (SAMS; Johnson, Gooding & Tarrier, 2008).  Three types of positive self-appraisals considered as important in buffering individuals from suicidal thoughts in the face of stressful life events (Johnson et al., 2008). These are appraisals of the individual’s ability to cope with emotions, solve problems, and gain social support. 12 items tool reflecting these areas. Responses rated on a 5 point scale from ‘strongly disagree’ to ‘strongly agree’. Preliminary psychometric properties reported by Johnson et al. (2010). | 2 independent studies:  Carandang et al. 2019  Li et al. 2015 |

**Supplementary Table 3.** National Heart, Lung and Blood Institute (NIH) Quality Assessment Tool for Observational Cohort and Cross-sectional Studies (NIH, 2020)

| **Study:** | | | **Date:** |
| --- | --- | --- | --- |
|  | | |  |
| **Criteria** | **Yes** | **No** | **Other (CD, NR, NA)*** |
| 1. Was the research question or objective in this paper clearly stated? |  |  |  |
| 2. Was the study population clearly specified and defined? |  |  |  |
| 3. Was the participation rate of eligible persons at least 50%? |  |  |  |
| 4. Were all the subjects selected or recruited from the same or similar populations (including the same time period)? Were inclusion and exclusion criteria for being in the study prespecified and applied uniformly to all participants? |  |  |  |
| 5. Was a sample size justification, power description, or variance and effect estimates provided? |  |  |  |
| 6. For the analyses in this paper, were the exposure(s) of interest measured prior to the outcome(s) being measured? |  |  |  |
| 7. Was the timeframe sufficient so that one could reasonably expect to see an association between exposure and outcome if it existed? |  |  |  |
| 8. For exposures that can vary in amount or level, did the study examine different levels of the exposure as related to the outcome (e.g., categories of exposure, or exposure measured as continuous variable)? |  |  |  |
| 9. Were the exposure measures (independent variables) clearly defined, valid, reliable, and implemented consistently across all study participants? |  |  |  |
| 10. Was the exposure(s) assessed more than once over time? |  |  |  |
| 11. Were the outcome measures (dependent variables) clearly defined, valid, reliable, and implemented consistently across all study participants? |  |  |  |
| 12. Were the outcome assessors blinded to the exposure status of participants? |  |  |  |
| 13. Was loss to follow-up after baseline 20% or less? |  |  |  |
| 14. Were key potential confounding variables measured and adjusted statistically for their impact on the relationship between exposure(s) and outcome(s)? |  |  |  |

CD=cannot determine; NA=not applicable; NR=not reported

| **Quality Rating (Good, Fair, or Poor)** | |
| --- | --- |
| Rater #1 initials: |  |
| Rater #2 initials: |  |
| Additional Comments (If POOR, please state why): |  |

**Guidance for Assessing the Quality of Observational Cohort and Cross-Sectional Studies**

The guidance document below is organized by question number from the tool for quality assessment of observational cohort and cross-sectional studies.

**Question 1. Research question**

Did the authors describe their goal in conducting this research? Is it easy to understand what they were looking to find? This issue is important for any scientific paper of any type. Higher quality scientific research explicitly defines a research question.

**Questions 2 and 3. Study population**

Did the authors describe the group of people from which the study participants were selected or recruited, using demographics, location, and time period? If you were to conduct this study again, would you know who to recruit, from where, and from what time period? Is the cohort population free of the outcomes of interest at the time they were recruited?

An example would be men over 40 years old with type 2 diabetes who began seeking medical care at Phoenix Good Samaritan Hospital between January 1, 1990 and December 31, 1994. In this example, the population is clearly described as: (1) who (men over 40 years old with type 2 diabetes); (2) where (Phoenix Good Samaritan Hospital); and (3) when (between January 1, 1990 and December 31, 1994). Another example is women ages 34 to 59 years of age in 1980 who were in the nursing profession and had no known coronary disease, stroke, cancer, hypercholesterolemia, or diabetes, and were recruited from the 11 most populous States, with contact information obtained from State nursing boards.

In cohort studies, it is crucial that the population at baseline is free of the outcome of interest. For example, the nurses' population above would be an appropriate group in which to study incident coronary disease. This information is usually found either in descriptions of population recruitment, definitions of variables, or inclusion/exclusion criteria.

You may need to look at prior papers on methods in order to make the assessment for this question. Those papers are usually in the reference list.

If fewer than 50% of eligible persons participated in the study, then there is concern that the study population does not adequately represent the target population. This increases the risk of bias.

**Question 4. Groups recruited from the same population and uniform eligibility criteria**

Were the inclusion and exclusion criteria developed prior to recruitment or selection of the study population? Were the same underlying criteria used for all of the subjects involved? This issue is related to the description of the study population, above, and you may find the information for both of these questions in the same section of the paper.

Most cohort studies begin with the selection of the cohort; participants in this cohort are then measured or evaluated to determine their exposure status. However, some cohort studies may recruit or select exposed participants in a different time or place than unexposed participants, especially retrospective cohort studies–which is when data are obtained from the past (retrospectively), but the analysis examines exposures prior to outcomes. For example, one research question could be whether diabetic men with clinical depression are at higher risk for cardiovascular disease than those without clinical depression. So, diabetic men with depression might be selected from a mental health clinic, while diabetic men without depression might be selected from an internal medicine or endocrinology clinic. This study recruits groups from different clinic populations, so this example would get a "no."

However, the women nurses described in the question above were selected based on the same inclusion/exclusion criteria, so that example would get a "yes."

**Question 5. Sample size justification**

Did the authors present their reasons for selecting or recruiting the number of people included or analyzed? Do they note or discuss the statistical power of the study? This question is about whether or not the study had enough participants to detect an association if one truly existed.

A paragraph in the methods section of the article may explain the sample size needed to detect a hypothesized difference in outcomes. You may also find a discussion of power in the discussion section (such as the study had 85 percent power to detect a 20 percent increase in the rate of an outcome of interest, with a 2-sided alpha of 0.05). Sometimes estimates of variance and/or estimates of effect size are given, instead of sample size calculations. In any of these cases, the answer would be "yes."

However, observational cohort studies often do not report anything about power or sample sizes because the analyses are exploratory in nature. In this case, the answer would be "no." This is not a "fatal flaw." It just may indicate that attention was not paid to whether the study was sufficiently sized to answer a prespecified question–i.e., it may have been an exploratory, hypothesis-generating study.

**Question 6. Exposure assessed prior to outcome measurement**

This question is important because, in order to determine whether an exposure causes an outcome, the exposure must come before the outcome.

For some prospective cohort studies, the investigator enrolls the cohort and then determines the exposure status of various members of the cohort (large epidemiological studies like Framingham used this approach). However, for other cohort studies, the cohort is selected based on its exposure status, as in the example above of depressed diabetic men (the exposure being depression). Other examples include a cohort identified by its exposure to fluoridated drinking water and then compared to a cohort living in an area without fluoridated water, or a cohort of military personnel exposed to combat in the Gulf War compared to a cohort of military personnel not deployed in a combat zone.

With either of these types of cohort studies, the cohort is followed forward in time (i.e., prospectively) to assess the outcomes that occurred in the exposed members compared to nonexposed members of the cohort. Therefore, you begin the study in the present by looking at groups that were exposed (or not) to some biological or behavioral factor, intervention, etc., and then you follow them forward in time to examine outcomes. If a cohort study is conducted properly, the answer to this question should be "yes," since the exposure status of members of the cohort was determined at the beginning of the study before the outcomes occurred.

For retrospective cohort studies, the same principal applies. The difference is that, rather than identifying a cohort in the present and following them forward in time, the investigators go back in time (i.e., retrospectively) and select a cohort based on their exposure status in the past and then follow them forward to assess the outcomes that occurred in the exposed and nonexposed cohort members. Because in retrospective cohort studies the exposure and outcomes may have already occurred (it depends on how long they follow the cohort), it is important to make sure that the exposure preceded the outcome.

Sometimes cross-sectional studies are conducted (or cross-sectional analyses of cohort-study data), where the exposures and outcomes are measured during the same timeframe. As a result, cross-sectional analyses provide weaker evidence than regular cohort studies regarding a potential causal relationship between exposures and outcomes. For cross-sectional analyses, the answer to Question 6 should be "no."

**Question 7. Sufficient timeframe to see an effect**

Did the study allow enough time for a sufficient number of outcomes to occur or be observed, or enough time for an exposure to have a biological effect on an outcome? In the examples given above, if clinical depression has a biological effect on increasing risk for CVD, such an effect may take years. In the other example, if higher dietary sodium increases BP, a short timeframe may be sufficient to assess its association with BP, but a longer timeframe would be needed to examine its association with heart attacks.

The issue of timeframe is important to enable meaningful analysis of the relationships between exposures and outcomes to be conducted. This often requires at least several years, especially when looking at health outcomes, but it depends on the research question and outcomes being examined.

Cross-sectional analyses allow no time to see an effect, since the exposures and outcomes are assessed at the same time, so those would get a "no" response.

**Question 8. Different levels of the exposure of interest**

If the exposure can be defined as a range (examples: drug dosage, amount of physical activity, amount of sodium consumed), were multiple categories of that exposure assessed? (for example, for drugs: not on the medication, on a low dose, medium dose, high dose; for dietary sodium, higher than average U.S. consumption, lower than recommended consumption, between the two). Sometimes discrete categories of exposure are not used, but instead exposures are measured as continuous variables (for example, mg/day of dietary sodium or BP values).

In any case, studying different levels of exposure (where possible) enables investigators to assess trends or dose-response relationships between exposures and outcomes–e.g., the higher the exposure, the greater the rate of the health outcome. The presence of trends or dose-response relationships lends credibility to the hypothesis of causality between exposure and outcome.

For some exposures, however, this question may not be applicable (e.g., the exposure may be a dichotomous variable like living in a rural setting versus an urban setting, or vaccinated/not vaccinated with a one-time vaccine). If there are only two possible exposures (yes/no), then this question should be given an "NA," and it should not count negatively towards the quality rating.

**Question 9. Exposure measures and assessment**

Were the exposure measures defined in detail? Were the tools or methods used to measure exposure accurate and reliable–for example, have they been validated or are they objective? This issue is important as it influences confidence in the reported exposures. When exposures are measured with less accuracy or validity, it is harder to see an association between exposure and outcome even if one exists. Also as important is whether the exposures were assessed in the same manner within groups and between groups; if not, bias may result.

For example, retrospective self-report of dietary salt intake is not as valid and reliable as prospectively using a standardized dietary log plus testing participants' urine for sodium content. Another example is measurement of BP, where there may be quite a difference between usual care, where clinicians measure BP however it is done in their practice setting (which can vary considerably), and use of trained BP assessors using standardized equipment (e.g., the same BP device which has been tested and calibrated) and a standardized protocol (e.g., patient is seated for 5 minutes with feet flat on the floor, BP is taken twice in each arm, and all four measurements are averaged). In each of these cases, the former would get a "no" and the latter a "yes."

Here is a final example that illustrates the point about why it is important to assess exposures consistently across all groups: If people with higher BP (exposed cohort) are seen by their providers more frequently than those without elevated BP (nonexposed group), it also increases the chances of detecting and documenting changes in health outcomes, including CVD-related events. Therefore, it may lead to the conclusion that higher BP leads to more CVD events. This may be true, but it could also be due to the fact that the subjects with higher BP were seen more often; thus, more CVD-related events were detected and documented simply because they had more encounters with the health care system. Thus, it could bias the results and lead to an erroneous conclusion.

**Question 10. Repeated exposure assessment**

Was the exposure for each person measured more than once during the course of the study period? Multiple measurements with the same result increase our confidence that the exposure status was correctly classified. Also, multiple measurements enable investigators to look at changes in exposure over time, for example, people who ate high dietary sodium throughout the follow-up period, compared to those who started out high then reduced their intake, compared to those who ate low sodium throughout. Once again, this may not be applicable in all cases. In many older studies, exposure was measured only at baseline. However, multiple exposure measurements do result in a stronger study design.

**Question 11. Outcome measures**

Were the outcomes defined in detail? Were the tools or methods for measuring outcomes accurate and reliable–for example, have they been validated or are they objective? This issue is important because it influences confidence in the validity of study results. Also important is whether the outcomes were assessed in the same manner within groups and between groups.

An example of an outcome measure that is objective, accurate, and reliable is death–the outcome measured with more accuracy than any other. But even with a measure as objective as death, there can be differences in the accuracy and reliability of how death was assessed by the investigators. Did they base it on an autopsy report, death certificate, death registry, or report from a family member? Another example is a study of whether dietary fat intake is related to blood cholesterol level (cholesterol level being the outcome), and the cholesterol level is measured from fasting blood samples that are all sent to the same laboratory. These examples would get a "yes." An example of a "no" would be self-report by subjects that they had a heart attack, or self-report of how much they weigh (if body weight is the outcome of interest).

Similar to the example in Question 9, results may be biased if one group (e.g., people with high BP) is seen more frequently than another group (people with normal BP) because more frequent encounters with the health care system increases the chances of outcomes being detected and documented.

**Question 12. Blinding of outcome assessors**

Blinding means that outcome assessors did not know whether the participant was exposed or unexposed. It is also sometimes called "masking." The objective is to look for evidence in the article that the person(s) assessing the outcome(s) for the study (for example, examining medical records to determine the outcomes that occurred in the exposed and comparison groups) is masked to the exposure status of the participant. Sometimes the person measuring the exposure is the same person conducting the outcome assessment. In this case, the outcome assessor would most likely not be blinded to exposure status because they also took measurements of exposures. If so, make a note of that in the comments section.

As you assess this criterion, think about whether it is likely that the person(s) doing the outcome assessment would know (or be able to figure out) the exposure status of the study participants. If the answer is no, then blinding is adequate. An example of adequate blinding of the outcome assessors is to create a separate committee, whose members were not involved in the care of the patient and had no information about the study participants' exposure status. The committee would then be provided with copies of participants' medical records, which had been stripped of any potential exposure information or personally identifiable information. The committee would then review the records for prespecified outcomes according to the study protocol. If blinding was not possible, which is sometimes the case, mark "NA" and explain the potential for bias.

**Question 13. Follow-up rate**

Higher overall follow-up rates are always better than lower follow-up rates, even though higher rates are expected in shorter studies, whereas lower overall follow-up rates are often seen in studies of longer duration. Usually, an acceptable overall follow-up rate is considered 80 percent or more of participants whose exposures were measured at baseline. However, this is just a general guideline. For example, a 6-month cohort study examining the relationship between dietary sodium intake and BP level may have over 90 percent follow-up, but a 20-year cohort study examining effects of sodium intake on stroke may have only a 65 percent follow-up rate.

**Question 14. Statistical analyses**

Were key potential confounding variables measured and adjusted for, such as by statistical adjustment for baseline differences? Logistic regression or other regression methods are often used to account for the influence of variables not of interest.

This is a key issue in cohort studies, because statistical analyses need to control for potential confounders, in contrast to an RCT, where the randomization process controls for potential confounders. All key factors that may be associated both with the exposure of interest and the outcome–that are not of interest to the research question–should be controlled for in the analyses.

For example, in a study of the relationship between cardiorespiratory fitness and CVD events (heart attacks and strokes), the study should control for age, BP, blood cholesterol, and body weight, because all of these factors are associated both with low fitness and with CVD events. Well-done cohort studies control for multiple potential confounders.

**Some general guidance for determining the overall quality rating of observational cohort and cross-sectional studies**

The questions on the form are designed to help you focus on the key concepts for evaluating the internal validity of a study. They are not intended to create a list that you simply tally up to arrive at a summary judgment of quality.

Internal validity for cohort studies is the extent to which the results reported in the study can truly be attributed to the exposure being evaluated and not to flaws in the design or conduct of the study–in other words, the ability of the study to draw associative conclusions about the effects of the exposures being studied on outcomes. Any such flaws can increase the risk of bias.

Critical appraisal involves considering the risk of potential for selection bias, information bias, measurement bias, or confounding (the mixture of exposures that one cannot tease out from each other). Examples of confounding include co-interventions, differences at baseline in patient characteristics, and other issues throughout the questions above. High risk of bias translates to a rating of poor quality. Low risk of bias translates to a rating of good quality. (Thus, the greater the risk of bias, the lower the quality rating of the study.)

In addition, the more attention in the study design to issues that can help determine whether there is a causal relationship between the exposure and outcome, the higher quality the study. These include exposures occurring prior to outcomes, evaluation of a dose-response gradient, accuracy of measurement of both exposure and outcome, sufficient timeframe to see an effect, and appropriate control for confounding–all concepts reflected in the tool.

Generally, when you evaluate a study, you will not see a "fatal flaw," but you will find some risk of bias. By focusing on the concepts underlying the questions in the quality assessment tool, you should ask yourself about the potential for bias in the study you are critically appraising. For any box where you check "no" you should ask, "What is the potential risk of bias resulting from this flaw in study design or execution?" That is, does this factor cause you to doubt the results that are reported in the study or doubt the ability of the study to accurately assess an association between exposure and outcome?

The best approach is to think about the questions in the tool and how each one tells you something about the potential for bias in a study. The more you familiarize yourself with the key concepts, the more comfortable you will be with critical appraisal. Examples of studies rated good, fair, and poor are useful, but each study must be assessed on its own based on the details that are reported and consideration of the concepts for minimizing bias.

**Supplementary Table 4.** Data Extraction Form, adapted from Joanna Brigg’s Institute (JBI) Reviewer's Manual (Aromataris & Munn, 2020)

| **Study details** | | | |
| --- | --- | --- | --- |
| Author(s): |  | | |
| Year: |  | | |
| Journal: |  | | |
| Title: |  | | |
| **Study method/characteristics** | | | |
| **Study design** –the type of study e.g., cohort study/cross-sectional study | |  | |
| **Setting** – e.g., hospital / community | |  | |
| **Sample size** | |  | |
| **Participants** - age, sex, country/location, diagnosis other relevant info | |  | |
| **Recruitment procedures** | |  | |
| **Follow-up or study duration** | |  | |
| **Exposure(s) of interest** (Independent variable) – type, frequency, duration | |  | |
| **Variables of interest & measurement** | | Outcome variable | Secondary variable(s) (independent) |
| Outcomes – the primary outcome measured and where relevant includes associated secondary outcomes. | |  |  |
| Outcome measurements – describe the scales or tools used to measure the outcomes. | |  |  |
| **Data analysis methods** including statistical technique, adjustment for confounding factors, etc. | |  | |
| **Study results / appropriate measures** | |  | |
| - E.g., Correlation coefficient r - Regression coefficient standardized β - Odds ratio - P value & 95% Confidence Intervals   t statistic  F statistic | |  | |
| **Reviewer’s comments** | |  | |

**Supplementary Table 5.** Included studies

| **Study** | **Country & design** | **n** | **Setting** | **Mean age** | **% Female** | **Resilience Measure** | **Resilience conceptualization** | **Factors included in meta-analysis** | **Quality rating** |
| --- | --- | --- | --- | --- | --- | --- | --- | --- | --- |
| Baldwin et al. (2011) | United States  Cross-sectional | 52 | Community | 74 | 65 | Ego Resiliency Scale (Block & Kremen, 1996) | Trait resilience | Psychological distress  Optimism | Poor |
| Bartley et al. (2019) | United States  Cross-sectional | 60 | Community | 68.1 | 56.7 | Brief Resilience Scale (Smith et al., 2008) | Resilience as coping process | Age  Gender  Race  Education  Marital status  Employment  Income | Good |
| Carandang et al. (2019) | Philippines  Cross-sectional | 1021 | Community | M: 67.3  F: 67.9 | 68.5 | Resilience Appraisal Scale (Johnson et al., 2010) | Resilience as coping process | Depressive symptoms | Good |
| Freitag & Schmidt (2016) | Germany  Cross-sectional | 210 | Community | 75.3 | 62.4 | The Resilience Scale (Wagnild & Young, 1993) | Trait Resilience | Depressive symptoms  QoL  Social support  Self-efficacy | Fair |
| Fullen et al. (2018) | United States  Cross-sectional | 200 | Independent living senior housing communities | 73.7 | 84 | Brief Resilience Scale (Smith et al., 2008) | Resilience as coping process | Age  Gender  Race  Education  Successful aging  Life satisfaction  Self-rated physical health  Psychological/mental wellbeing | Fair |
| Hardy et al. (2004) | United States  Cross-sectional | 546 | Community | 73.72 | 64 | Hardy-Gill Resilience Scale (Hardy et al., 2004) | Resilience as coping process | Depressive symptoms  Perceived stressfulness of event | Good |
| Jeste et al. (2013) | United States  Cross-sectional | 1006 | Community | 77.3 | NR | Connor Davidson Resilience Scale (Connor & Davidson, 2008) | Trait resilience | Successful aging | Good |
| Jeste et al. (2019) | United States  Cross-sectional | 104 | Continuing care senior housing community | 83.6 | 67 | Connor Davidson Resilience Scale (Connor & Davidson, 2008) | Trait resilience | Self-rated physical health | Poor |
| King & Richardson (2016) | United States  Cross-sectional | 316 | Community | 57.78 | 0 | The Resilience Scale (Wagnild & Young, 1993) | Trait resilience | Marital status  Income  Social support  Support from family  Support from friends  Stigma | Fair |
| Kuwert et al. (2014) | United States Cross-sectional | 2025 | Community | 71 | 3.8 | Connor Davidson Resilience Scale-10 (Connor & Davidson, 2008) | Trait resilience | Loneliness | Good |
| Lamond et al. (2008) | United States  Cross-sectional | 1395 | Community | 72.7 | 100 | Connor Davidson Resilience Scale (Connor & Davidson, 2008) | Trait resilience | Age  Education  Successful aging  Self-rated physical health (SF-36)  Self-rated mental health (SF-6)  Social engagement  Optimism  Cognitive functioning | Fair |
| Lee et al. (2008) | United States  Cross-sectional | 200 | Community | 72.5 | 100 | The Resilience Scale (Wagnild & Young, 1993) | Trait resilience | Age  Optimism  Spirituality | Fair |
| Li et al. (2015) | Singapore  Cross-sectional | 162 | Community | 72.2 | 75.9 | Resilience Appraisal Scale (Johnson et al., 2010) | Resilience as coping process | Age  Gender  Education  Depressive symptoms  QoL  Social support  Loneliness | Fair |
| Liddell & Ferreira (2019) | United States Cross-sectional | 5713 | Community | 73.3 | 63.7 | The Pearlin Mastery Scale (Pearlin & Schooler, 1978) | Tait resilience | Age  Gender  Marital status  Self-rated general health  Self-rated physical health  Self-rated mental health  Depression  Life satisfaction | Fair |
| Lim et al. (2015) | China  Cross-sectional | 385 | Community | 72.1 | 58.1 | Connor Davidson Resilience Scale (Connor & Davidson, 2008) | Trait resilience | Depressive symptoms | Good |
| Lu et al. (2017) | China  Cross-sectional | 474 | Community | 69.3 | 53 | Connor Davidson Resilience Scale (Connor & Davidson, 2008) | Trait resilience | Depressive symptoms  Self-rated health (SF-12)  Self-rated physical health (SF-12)  Self-rated mental health (SF-12)  Optimism | Fair |
| Manning et al. (2016) | United States  Longitudinal | 10753 | Community | 68.6 | 59 | Simplified Resilience Score (Zeng & Shen, 2010) based on Wagnild & Young (1993) Resilience Scale | Trait resilience | ADL limitations | Poor |
| Martin et al. (2015b) | United States  Cross-sectional | N=1006  YO:365  OO:641 | Community | YO:63.3  OO:85.3 | YO:49  OO:48.3 | Connor Davidson Resilience Scale (Connor & Davidson, 2008) | Trait resilience | Successful aging | Fair |
| McClain et al. (2018) | United States  Cross-sectional | 58 | Community | 74.5 | 74.1 | The Resilience Scale (Wagnild & Young, 1993) | Trait resilience | Self-rated physical health (SF-36)  Self-efficacy  Social support | Fair |
| McKibbin et al. (2016) | United States  Cross-sectional | 198 | Community-rural | 73.7 | 52.5 | Connor Davidson Resilience Scale (Connor & Davidson, 2008) | Trait resilience | Self-rated physical health (SF-12)  Self-rated mental health (SF-12)  Family network size  Friend network size | Fair |
| Mehta et al. (2008) | United States  Cross-sectional | 105 | Community | 79.3 | 80 | Hardy-Gill Resilience Scale (Hardy et al 2004) | Resilience as coping process | Depressive symptoms | Fair |
| Moe et al. (2013) | Norway  Cross-sectional | 120 | Community – home or sheltered housing | 87.5 | 65.8 | The Resilience Scale (Wagnild & Young, 1993) | Trait resilience | Self-rated physical health (SF-36)  Self-rated mental health (SF-36)  Self-transcendence  Purpose in life  Sense of coherence | Fair |
| Montross et al. (2006) | United States Cross-sectional | 205 | Continuing care retirement communities | 80.4 | 60 | Connor Davidson Resilience Scale (Connor & Davidson, 2008) | Trait resilience | Successful aging | Fair |
| Moore et al. (2015) | United States  Cross-sectional | 1006 | Community | 77 | NR | Connor Davidson Resilience Scale (Connor & Davidson, 2008) | Trait resilience | Successful aging  Self-rated physical health (SF-36)  Self-rated mental health (SF-36)  Social support  Psychological distress | Fair |
| Nygren et al. (2005) | Sweden Cross-sectional | 125 | Community | NR (85+) | 69 | The Resilience Scale (Wagnild & Young, 1993) | Trait resilience | Self-rated physical health (SF-36)  Self-rated mental health (SF-36)  Self-transcendence  Purpose in life  Sense of coherence | Fair |
| Ong et al. (2006) | United States  Cross-sectional | 27 | Community | 72.1 | 48 | Ego Resiliency Scale (Block & Kremen, 1996) | Trait resilience | Psychological distress  Positive daily emotions  Negative daily emotions | Fair |
|  | United States  Cross-sectional | 40 | Community | 75.5 | 50 | Dispositional Resilience Scale (Bartone et al., 1989) | Trait resilience | Psychological distress  Positive daily emotions  Negative daily emotions | Fair |
|  | United States  Cross-sectional | 34 | Community | 71.9 | 100 | Dispositional Resilience Scale (Bartone et al., 1989) | Trait resilience | Psychological distress  Positive daily emotions  Negative daily emotions | Fair |
| de Paula Couto et al. (2011) | Brazil  Cross-sectional | 111 | Community | 68.6 | 83 | The Resilience Scale (Wagnild & Young, 1993) | Trait Resilience | Age  Gender  Psychological/mental wellbeing | Fair |
| Polson et al. (2018) | United States  Cross-sectional | 64 | Community | 72.7 | 67 | The Resilience Scale (Wagnild & Young, 1993) | Trait resilience | Age  Gender  Race  Self-rated mental health  Social support  Cognitive functioning  Spirituality | Fair |
| Rossi et al. (2007) | United States  Cross-sectional | 55 | Community | 71.5 | 100 | Dispositional Resilience Scale (Bartone et al., 1989) | Trait resilience | Age  Perceived stressfulness of event  Life satisfaction | Fair |
| Scelzo et al. (2018) | Italy  Cross-sectional | N=80  YO: 51  OO: 29 | Community | NR  YO:51-75  OO:90-101 | NR | Connor Davidson Resilience Scale (Connor & Davidson, 2008) | Trait resilience | Age  Self-rated physical health (SF-12)  Self-rated mental health (SF-12) | Fair |
| Schure et al. (2013) | United States  Cross-sectional | 185 | Community | 68.7 | 69.2 | 10-item abbreviated version of Connor-Davidson Resilience Scale (CD-RISC, Campbell-Sills & Stein, 2007; Connor & Davidson, 2003 | Trait resilience | Self-rated physical health (SF-8)  Self-rated mental health (SF-8) | Fair |
| Silverman et al. (2015) | United States  Longitudinal | 1594 | Community | 56 | 64 | Connor Davidson Resilience Scale (Connor & Davidson, 2008) | Trait resilience | Education  Income  Depressive symptoms  Physical functioning  Social engagement | Good |
| Smith (2009) | United States  Cross-sectional | 158 | Community | NR (65+) | 76.6 | Connor Davidson Resilience Scale (Connor & Davidson, 2008) | Trait resilience | Self-rated health | Good |
| Smith (2012) | United States  Cross-sectional | 158 | Community | 75.2 | 76 | Connor Davidson Resilience Scale (Connor & Davidson, 2008) | Trait resilience | Depressive symptoms  Stigma | Poor |
| Torma et al. (2013) | United States  Cross-sectional | 224 | Community | 62.1 | 94 | The Resilience Scale (Wagnild & Young, 1993) | Trait resilience | Age  Depressive symptoms  Physical function  Social support | Good |
| Vahia et al. (2011) | United States  Cross-sectional | 1942 | Community | 73 | 100 | Connor Davidson Resilience Scale (Connor & Davidson, 2008) | Trait resilience | Spirituality | Good |
| Wagnild (2003)  Low income | United States  Cross-sectional | 43 | Community | 73.4 | 83.8 | The Resilience Scale (Wagnild & Young, 1993) | Trait resilience | Life satisfaction  Self-rated health  Morale | Poor |
| High income | United States  Cross-sectional | 176 | Community | 69.5 | 49.4 | The Resilience Scale (Wagnild & Young, 1993) | Trait resilience | Life satisfaction  Self-rated health  Morale | Poor |
| Low income | United States  Cross-sectional | 161 | Community | 74.9 | 85.1 | The Resilience Scale (Wagnild & Young, 1993) | Trait resilience | Life satisfaction  Self-rated health  Morale | Poor |
| High income | United States  Cross-sectional | 232 | Community | 69.1 | 44 | The Resilience Scale (Wagnild & Young, 1993) | Trait resilience | Self-rated health  Health promoting lifestyle | Poor |
| Low income | United States  Cross-sectional | 112 | Community | 74.9 | 83.9 | The Resilience Scale (Wagnild & Young, 1993) | Trait resilience | Self-rated health  Health promoting lifestyle | Poor |
| Wagnild & Torma (2013) | United States  Cross-sectional | 25 | Community | 75.7 | 100 | The Resilience Scale (Wagnild & Young, 1993) | Trait resilience | Self-rated health  Health promoting lifestyle | Poor |
| Wagnild & Young (1993) | United States  Cross-sectional | 810 | Community | 71.1 | 62.3 | The Resilience Scale (Wagnild & Young, 1993) | Trait resilience | Depressive symptoms  Life satisfaction  Self-rated health  Morale | Good |
| Wallace et al. (2001) | United States  Cross-sectional | range: 367-421 | Community | 75 | 77 | Dispositional Resilience Scale (Bartone et al., 1989) | Trait Resilience | Support from family  Support from friends | Poor |
| Wells (2009) | United States  Cross-sectional | 106 | Community | 75 | 54 | The Resilience Scale (Wagnild & Young, 1993) | Trait resilience | Self-rated physical health (SF-12)  Self-rated mental health (SF-12)  Social support  Friend network size | Fair |
| Wells (2010) | United States  Cross-sectional | 277 | Community | 75 | 53.4 | The Resilience Scale (Wagnild & Young, 1993) | Trait resilience | Self-rated physical health (SF-12)  Self-rated mental health (SF-12)  Family network size  Friend network size | Fair |
| You & Park (2017) | South Korea  Cross-sectional | 2034 | Community | 74.5 | 59 | Connor Davidson Resilience Scale (Connor & Davidson, 2008) | Trait resilience | Risk of suicidal behavior | Good |

**Quality ratings**

**Good (12)**: Bartley et al. (2019); Carandang et al. (2019); Hardy et al. (2004); Jeste et al. (2013); Kuwert et al. (2014); Lim et al. (2015); Silverman et al. (2015); Smith (2009); Torma et al. (2013); Vahia et al. (2014); Wagnild & Young (1993); You & Park (2017)

**Fair (24)**: Couto et al. (2011); Freitag & Schmidt (2016); Fullen et al. (2018); King & Richardson (2016); Lamond et al. (2008); Lee et al. (2008); Li et al (2015); Liddell & Fereira (2019); Lu et al. (2017); Martin et al. (2015b); McClain et al. (2018); McKibbin et al. (2016); Mehta et al. (2008); Moe et al. (2013); Montross et al. (2006); Moore et al. (2015); Nygren et al. (2005); Ong et al. (2006); Polson et al. (2018); Rossi et al. (2007); Scelzo et al. (2018); Schure et al. (2013); Wells (2009); Wells (2010)

**Poor (7)**: Baldwing et al. (2011); Jeste et al. (2019); Manning et al. (2016); Smith (2012); Wagnild (2003); Wagnild & Torma (2013); Wallace et al. (2001)

**Design:** longitudinal 2; cross-sectional 41

**Countries**

| USA: 33  China: 2 | Brazil: 1  Germany: 1 | Italy: 1  Norway: 1 | Philippines: 1  Singapore: 1 | South Korea: 1  Sweden: 1 |
| --- | --- | --- | --- | --- |

**Setting**

Community: 39

Independent living senior housing communities: 3

Community – home or sheltered housing: 1

**Supplementary Table 6.** Articles excluded at the full-text screening stage

| **Primary reason for exclusion** | **Article** |
| --- | --- |
| Mean age ˂ 55 (n = 17) | Bernstein et al. (2017); Beutel et al. (2009); Brennan et al. (2017); Campbell-Sills et al. (2009); Driver et al. (2016); Eshel et al. (2018); Guest et al. (2015); Holden et al. (2013); Kilic et al. (2013); Marciano et al. (2019); Morote et al. (2017); Pekenham et al. (2018); Reyes et al. (2019); Sharpley et al. (2014); Spies & Seedat, (2014); Topel et al. (2019); Tugade et al. (2004) |
| No age data reported (n=1) | Wisco et al. (2014) |
| Non-standardized measure of resilience (n = 10) | Emlet et al. (2017); Hardy et al. (2002); Liebenberg & Moore (2018); Netuveli et al. (2008); Sawyer & Allman (2010); Shen & Zeng (2011); Smith & Hollinger-Smith (2015); Talsma (1996); Yang & Wen (2015); Zeng & Shen (2010) |
| Measure of physical resilience (n = 1) | Klinedinst & Resnick (2014) |
| No resilience measure (n = 5) | Cunha et al. (2017); Elby et al. (1996); Heisel et al. (2020); Pietrzak et al. (2014); Roos & Havens (1991); |
| Intervention study (n = 1) | Sun & Buys (2014) |
| Setting (n = 1) | Holmes et al. (2019) |
| Language (n = 3) | Min et al. (2017); Lei et al. (2018); Serrano-Parra et al. (2012) |
| Dissertation, findings in peer reviewed paper (included) (n = 1) | Fullen (2016) |
| Unable to source (despite, where possible, contacting authors) (n = 6) | Bane (1998); Boseman (2001); Johnson (2002); Lee et al. (2018); Wallace (1999) |
| Data not suitable for computing the desired effect size (n = 13) | Clark et al. (2019); Fullen & Granello (2018); Martins et al. (2011); Gooding et al. (2012); Lamet et al. (2008); Lau et al. (2010); Lau et al. (2018); Mertens et al. (2012); Phillips et al. (2016); Siu et al. (2018); Stewart et al. (2019); Tomás et al. (2012); Vahia et al. (2010) |

**Supplementary Table 7.** Personal factors associated with resilience identified across studies meeting inclusion criteria

|  | **Factor** | **Reported in** |
| --- | --- | --- |
| 1 | ADL limitations* | Manning et al. (2016); Polson et al. (2018) |
| 2 | Age* | Bartley et al. (2019); Fullen et al. (2018); Lamond et al. (2008); Lee et al. (2008); Li et al. (2015); Liddell & Ferreir (2019); ^1^*McKibbin et al. (2016)*; de Paula Couto et al. (2011); Polson et al (2018); Rossi et al. (2007); Scelzo et al. (2018) (YO); Scelzo et al. (2018) (OO); Torma et al. (2013); ^2,3^*Zeng & Shen, (2010)* |
| 3 | Cognitive functioning* | Lamond et al. (2008); Polson et al. (2018) |
| 4 | Depressive symptoms* | Carandang et al. (2019); Freitag & Schmidt (2016); Hardy et al. (2004); Li et al. (2015); Lim et al. (2015); Lu et al. (2017); Mehta et al. (2008); *^2^Schure et al. (2013)*; Silverman et al. (2015); Smith (2012); Torma et al. (2013); ^2^*Vahia et al. (2010)*; Wagnild & Young (1993) |
| 5 | Gender* | Bartley et al. (2019); Fullen et al. (2018); Li et al. (2015); Liddell & Ferreira (2019); ^1^*McKibbin et al. (2016)*; de Paula Couto et al. (2011); Polson et al. (2018) |
| 6 | Health promoting lifestyle* | Wagnild (2003) (2 independent samples); Wagnild & Torma (2013) |
| 7 | Life satisfaction* | Fullen et al. (2018); Rossi et al. (2007); Wagnild (2003) (low income); Wagnild (2003) (high income); Wagnild (2003) (low income); Wagnild & Young (1993) |
| 8 | Loneliness* | Kuwert et al. (2014); Li et al. (2015) |
| 9 | Morale* | Wagnild (2003) (3 independent samples); Wagnild & Young (1993) |
| 10 | Optimism* | Baldwin et al. (2011); Lamond et al. (2008); Lee et al. (2008); Lu et al. (2017) |
| 11 | Physical functioning* | ^2^*Mertens et al. (2012)*; Silverman et al. (2015); Torma et al. (2011) |
| 12 | Positive daily emotions* | Ong et al. (2006) (3 independent samples) |
| 13 | Negative daily emotions* | Ong et al. (2006) (3 independent samples) |
| 14 | Psychological distress* | Baldwin et al. (2011); Moore et al. (2015); Ong et al. (2006) (3 independent samples) |
| 15 | Psychological wellbeing* | Fullen et al. (2018); de Paula Couto et al. (2011) |
| 16 | Purpose in life* | Moe et al. (2013); Nygren et al. (2005) |
| 17 | Quality of life* | Freitag & Schmidt (2016); Li et al. (2015) |
| 18 | Self-efficacy* | Freitag & Schmidt (2016); McClain et al. (2018) |
| 19 | Self-rated general health* | Liddell & Ferreira (2019); Lu et al. (2017); Smith (2009); Wagnild (2003) (low income); Wagnild (2003) (high income); Wagnild (2003) (low income); Wagnild (2003) (high income); Wagnild (2003) (low income); Wagnild & Torma (2013); Wagnild & Young (1993) |
| 20 | Self-rated mental health* | Lamond et al. (2008); Liddell & Ferreira (2019); Lu et al. (2017); McKibbin et al. (2016); Moe et al. (2013); Moore et al. (2015); Nygren et al. (2005); Polson et al. (2018); Scelzo et al. (2018) (YO); Scelzo et al. (2018) (OO); Schure et al. (2013); Wells (2009); Wells (2010) |
| 21 | Self-rated physical health* | Fullen et al. (2018); ^1^*Jeste et al. (2019)*; Lamond et al. (2008); Liddell & Ferreira (2019); Lu et al. (2017); McClain et al. (2018); McKibbin et al. (2016); Moe et al. (2013); Moore et al. (2015); Nygren et al. (2005); Scelzo et al. (2018) (YO); Scelzo et al. (2018) (OO); Schure et al. (2013); Wells (2009); Wells (2010) |
| 22 | Self-transcendence* | Moe et al. (2013); Nygren et al. (2005) |
| 23 | Sense of coherence* | Moe et al. (2013); Nygren et al. (2005) |
| 24 | Social engagement* | Lamond et al. (2008); *^1^Phillips et al. (2016);* Silverman et al. (2015) |
| 25 | Spirituality* | Lee et al. (2008); Polson et al. (2018); Vahia et al. (2011) |
| 26 | Successful aging* | Fullen et al. (2018); Jeste et al. (2013); Lamond et al. (2008*)*; *^4^Martin et al. (2015b) (YO); ^4^Martin et al. (2015b) (OO)*; Montross et al. (2006); ^4^*Moore et al. (2015)*; ^2^*Stewart et al. (2019)* |
| 27 | Suicidal behavior risk* | You & Park (2017) (men); You & Park (2017) (women) |
| 28 | Willingness to seek help for depressive symptoms | ^4^*Smith (2009)*; ^4^*Smith (2012)* |
| 29 | ADL Independent | Hardy et al. (2004) |
| 30 | Anxiety | Freitag & Schmidt (2016) |
| 31 | Childhood physical abuse | Phillips et al. (2016) |
| 32 | Childhood social adversity | Phillips et al. (2016) |
| 33 | Childhood economic adversity | Phillips et al. (2016) |
| 34 | Comorbidity | Torma et al. (2013) |
| 35 | Chronic pain | Schure et al. (2013) |
| 36 | Current mood | Smith (2009) |
| 37 | Frailty | Freitag & Schmidt (2016) |
| 38 | Happiness | Fullen et al. (2018) |
| 39 | Hope | Polson et al. (2018) |
| 40 | Internal health locus of control | King & Richardson (2016) |
| 41 | IADL limitations | Manning et al. (2016) |
| 42 | Internalized homophobia | King & Richardson (2016) |
| 43 | Pain duration | Bartley et al. (2019) |
| 44 | Physical activity | Torma et al. (2011) |
| 45 | Self-esteem | Lee et al. (2008) |
| 46 | Self-perceived oral health | Martins et al. (2011) |
| 47 | Strength | Lu et al. (2017) |
| 48 | Tenacity | Lu et al. (2017) |

*data available for meta-analysis

*Italics*: data excluded from meta-analysis [reasons for exclusion: ^1^data not suitable for computing the required effect size; ^2^categorical variable; ^3^non-standardised measurement of resilience; ^4^non-independent samples]

YO – young-old (as defined by authors); OO – old-old (as defined by authors)

**Supplementary Table 8.** Contextual factors associated with resilience identified across studies meeting inclusion criteria

|  | Factor | Reported in |
| --- | --- | --- |
| 1 | Education* | Bartley et al. (2019); Fullen et al. (2018); Lamond et al. (2008); Li et al. (2015); ^1^*McKibbin et al. (2016)*; Silverman et al. (2015); ^2,3^*Zeng & Shen. (2010)* |
| 2 | Income* | Bartley et al. (2019); King & Richardson (2016); ^1^*Philips et al. (2016)*; Silverman et al. (2015) |
| 3 | Marital status* | Bartley et al. (2019); King & Richardson (2016); Liddell & Ferreira (2019); ^1^*McKibbin et al. (2016)*; ^2,3^*Zeng & Shen (2010)* |
| 4 | Race* | Bartley et al. (2019); Fullen et al. (2018); Polson et al. (2018) |
| 5 | Family network size* | McKibbin et al. (2016); Wells (2010) |
| 6 | Friend network size* | McKibbin et al. (2016); Wells (2009); Wells (2010) |
| 7 | Perceived stressfulness of the event* | Hardy et al. (2004); Rossi et al. (2007) |
| 8 | Social support* | Freitag & Schmidt (2016); King & Richardson (2016); Li et al. (2015); McClain et al. (2018); Moore et al. (2015); Polson et al. (2018); Torma et al. (2013); Wells (2009) |
| 90 | Stigma* | King & Richardson (2016); Smith (2012) |
| 10 | Support from friends* | King & Richardson (2016); ^2^*Philips et al. (2016)*; Wallace et al. (2011) |
| 11 | Support from family* | King & Richardson (2016); ^2^*Philips et al. (2016)*; Wallace et al. (2011) |
| 12 | Living with others | *^2^Hardy et al. (2004)*; *^1^Liddell & Ferreira (2019)*; Li et al. (2015) |
| 13 | Cultural interdependency | Lee et al. (2008) |
| 14 | Discrimination | King & Richardson (2016) |
| 15 | Emotional support | Liddell & Ferreira (2019) |
| 16 | Employment | Bartley et al. (2019); *^1^McKibbin et al. (2016)* |
| 17 | Family functioning | Lu et al. (2017) |
| 18 | Length of marriage | Rossi et al. (2007) |
| 19 | Medical insurance | Fullen et al. (2018) |
| 20 | Perceived income adequacy | Li et al. (2015) |
| 21 | Race related stress | Baldwin et al. (2011) |
| 22 | Stressful events (number and intensity) | de Paula Couto et al. (2011) |
| 23 | Traumatic life events | Freitag & Schmidt (2016) |

*data available for meta-analysis

*Italics*: data excluded from meta-analysis [reasons for exclusion: ^1^data not suitable for computing the required effect size; ^2^categorical variable; ^3^non-standardised measurement of resilience]

**Supplementary Table 9.** Leave-one-out sensitivity analysis

| **Study omitted** | **r [95% CI]** | **r_p_** | **I^2^** |
| --- | --- | --- | --- |
| **Depressive symptoms** |  |  |  |
| Carandang et al. (2019) | -0.393 [-0.478,-0.301] | <0.001 | 91.40% |
| Freitag & Schmidt (2016) | -0.36 [-0.456,-0.254] | <0.001 | 95.30% |
| Hardy et al. (2004) | -0.375 [-0.473,-0.269] | <0.001 | 95.20% |
| Li et al. (2015) | -0.355 [-0.45,-0.253] | <0.001 | 95.30% |
| Lim et al. (2015) | -0.384 [-0.477,-0.284] | <0.001 | 95.00% |
| Lu et al. (2017) | -0.36 [-0.457,-0.254] | <0.001 | 95.30% |
| Mehta et al. (2008) | -0.378 [-0.472,-0.275] | <0.001 | 95.30% |
| Silverman et al. (2015) | -0.347 [-0.437,-0.25] | <0.001 | 90.60% |
| Smith (2012) | -0.39 [-0.476,-0.295] | <0.001 | 95.00% |
| Torma et al. (2013) | -0.351 [-0.444,-0.251] | <0.001 | 95.10% |
| Wagnild & Young (1993) | -0.369 [-0.468,-0.261] | <0.001 | 95.30% |
| **Pooled estimate** | **-0.369 [-0.459,-0.273]** | **<0.001** | **94.80%** |
| **Successful aging** |  |  |  |
| Fullen et al. (2018) | 0.273 [0.069,0.455] | 0.009 | 97.3% |
| Jeste et al. (2013) | 0.309 [0.154,0.449] | <0.001 | 86.9% |
| Lamond et al. (2008) | 0.170 [0.062,0.275] | 0.002 | 64.1% |
| Montross et al. (2006) | 0.243 [0.031,0.434] | 0.025 | 97.4% |
| **Pooled estimate** | **0.252 [0.096,0.396]** | **0.002** | **96.1%** |
| **Life satisfaction** |  |  |  |
| Fullen et al. (2018) | 0.333 [0.312,0.354] | <0.001 | 28.00% |
| Rossi et al. (2007) | 0.333 [0.312,0.353] | <0.001 | 21.80% |
| Wagnild (2003) (low income) | 0.332 [0.311,0.352] | <0.001 | 0.00% |
| Wagnild (2003) (high income) | 0.332 [0.311,0.353] | <0.001 | 23.80% |
| Wagnild (2003) (low income) | 0.333 [0.312,0.353] | <0.001 | 26.30% |
| Wagnild & Young (1993) | 0.35 [0.31,0.39] | <0.001 | 13.80% |
| Liddell & Ferreira (2019) | 0.356 [0.295,0.414] | <0.001 | 28.40% |
| **Pooled estimate** | **0.334 [0.313,0.354]** | **<0.001** | **15.10%** |
| **Self-rated physical health** |  |  |  |
| Fullen et al. (2018) | 0.186 [0.14,0.232] | <0.001 | 51.00% |
| Lamond et al. (2008) | 0.209 [0.161,0.256] | <0.001 | 50.90% |
| Liddell & Ferreira (2019) | 0.203 [0.149,0.256] | <0.001 | 57.60% |
| Lu et al. (2017) | 0.181 [0.139,0.222] | <0.001 | 38.70% |
| McClain et al. (2018) | 0.197 [0.148,0.245] | <0.001 | 60.20% |
| McKibbin et al. (2016) | 0.2 [0.149,0.25] | <0.001 | 60.20% |
| Moe et al. (2013) | 0.195 [0.145,0.243] | <0.001 | 59.30% |
| Moore et al. (2015) | 0.195 [0.141,0.247] | <0.001 | 56.70% |
| Nygren et al. (2005) | 0.203 [0.154,0.251] | <0.001 | 58.70% |
| Scelzo et al. (2018) (YO) | 0.197 [0.148,0.245] | <0.001 | 60.10% |
| Scelzo et al. (2018) (OO) | 0.196 [0.148,0.244] | <0.001 | 60.10% |
| Schure et al. (2013) | 0.206 [0.159,0.253] | <0.001 | 55.90% |
| Wells (2009) | 0.195 [0.145,0.244] | <0.001 | 59.60% |
| Wells (2010) | 0.195 [0.143,0.245] | <0.001 | 59.00% |
| **Pooled estimate** | **0.197 [0.15,0.244]** | **<0.001** | **56.90%** |
| **Self-rated mental health** |  |  |  |
| Lamond et al. (2008) | 0.35 [0.264,0.431] | <0.001 | 85.00% |
| Liddell & Ferreira (2019) | 0.377 [0.29,0.457] | <0.001 | 81.00% |
| Lu et al. (2017) | 0.358 [0.268,0.442] | <0.001 | 91.60% |
| McKibbin et al. (2016) | 0.354 [0.267,0.436] | <0.001 | 91.60% |
| Moe et al. (2013) | 0.363 [0.274,0.446] | <0.001 | 92.00% |
| Moore et al. (2015) | 0.363 [0.271,0.448] | <0.001 | 91.80% |
| Nygren et al. (2005) | 0.364 [0.275,0.447] | <0.001 | 92.00% |
| Polson et al. (2018) | 0.378 [0.297,0.453] | <0.001 | 91.90% |
| Scelzo et al. (2018) (YO) | 0.366 [0.279,0.446] | <0.001 | 92.00% |
| Scelzo et al. (2018) (OO) | 0.374 [0.292,0.45] | <0.001 | 92.00% |
| Schure et al. (2013) (adjusted) | 0.394 [0.331,0.455] | <0.001 | 91.00% |
| Wells (2009) | 0.348 [0.268,0.424] | <0.001 | 91.40% |
| Wells (2010) | 0.359 [0.269,0.443] | <0.001 | 91.80% |
| **Pooled estimate** | **0.365 [0.284,0.442]** | **<0.001** | **91.30%** |
| **Self-rated health total** |  |  |  |
| Liddell & Ferreira (2019) | 0.318 [0.266,0.37] | <0.001 | 38.10% |
| Lu et al. (2017) | 0.271 [0.211,0.329] | <0.001 | 68.80% |
| Smith (2009) | 0.297 [0.228,0.362] | <0.001 | 82.60% |
| Wagnild (2003) (low income) | 0.302 [0.239,0.363] | <0.001 | 82.40% |
| Wagnild (2003) (high income) | 0.285 [0.218,0.349] | <0.001 | 80.70% |
| Wagnild (2003) (low income)2 | 0.293 [0.224,0.358] | <0.001 | 82.20% |
| Wagnild (2003) (high income)2 | 0.29 [0.22,0.356] | <0.001 | 81.40% |
| Wagnild (2003) (low income)3 | 0.293 [0.225,0.358] | <0.001 | 82.40% |
| Wagnild & Torma (2013) | 0.287 [0.224,0.348] | <0.001 | 81.60% |
| Wagnild & Young (1993) | 0.301 [0.229,0.369] | <0.001 | 82.10% |
| **Pooled estimate** | **0.293 [0.231,0.354]** | **<0.001** | **80.70%** |
| **Social support** |  |  |  |
| Freitag & Schmidt (2016) | 0.257 [0.115,0.388] | <0.001 | 83.80% |
| King & Richardson (2016) | 0.275 [0.128,0.41] | <0.001 | 85.80% |
| Li et al. (2015) | 0.222 [0.136,0.306] | <0.001 | 62.60% |
| McClain et al. (2018) | 0.26 [0.123,0.388] | <0.001 | 85.40% |
| Moore et al. (2015) | 0.291 [0.149,0.42] | <0.001 | 82.50% |
| Polson et al. (2018) | 0.295 [0.166,0.413] | <0.001 | 85.30% |
| Torma et al. (2013) | 0.299 [0.169,0.419] | <0.001 | 84.00% |
| Wells (2009) | 0.283 [0.142,0.412] | <0.001 | 85.90% |
| **Pooled estimate** | **0.273 [0.149,0.389]** | **<0.001** | **83.60%** |
| **Friend network size** |  |  |  |
| McKibbin et al 2016 | 0.207 [0.109,0.302] | <0.001 | 0.00% |
| Wells 2009 | 0.239 [0.152,0.323] | <0.001 | 0.00% |
| Wells 2010 | 0.253 [0.144,0.356] | <0.001 | 0.00% |
| **Pooled estimate** | **0.232 [0.154,0.308]** | **<0.001** | **0.00%** |
| **Age** |  |  |  |
| Bartley et al. (2019) | 0.004 [-0.098,0.105] | 0.945 | 83.20% |
| Couto et al. (2011) | 0.009 [-0.099,0.117] | 0.868 | 83.60% |
| Fullen et al. (2018) | 0.003 [-0.102,0.108] | 0.952 | 80.60% |
| Lamond et al. (2008) | 0.038 [-0.073,0.148] | 0.5 | 85.00% |
| Lee et al. (2008) | 0.013 [-0.099,0.125] | 0.821 | 83.40% |
| Li et al. (2015) | 0.03 [-0.082,0.142] | 0.6 | 85.20% |
| Liddell & Ferreira (2019) | 0.04 [-0.071,0.149] | 0.482 | 80.70% |
| Polson et al. (2018) | 0.043 [-0.058,0.143] | 0.407 | 84.80% |
| Rossi et al. (2007) | 0.034 [-0.074,0.141] | 0.543 | 85.20% |
| Scelzo et al. (2018) (YO) | 0.041 [-0.061,0.142] | 0.427 | 84.80% |
| Scelzo et al. (2018) (OO) | 0.016 [-0.091,0.122] | 0.773 | 84.90% |
| Torma et al. (2013) | 0.003 [-0.102,0.108] | 0.957 | 79.30% |
| **Pooled estimate** | **0.023 [-0.079,0.125]** | **0.661** | **83.70%** |
| **Gender** |  |  |  |
| Bartley et al. (2019) | 0.049 [-0.031,0.127] | 0.23 | 45.60% |
| Couto et al. (2011) | 0.049 [-0.034,0.133] | 0.248 | 44.50% |
| Fullen et al. (2018) | 0.064 [-0.017,0.145] | 0.12 | 26.60% |
| Li et al. (2015) | 0.093 [0.051,0.135] | <0.001 | 0.00% |
| Liddell & Ferreira (2019) | 0.008 [-0.073,0.09] | 0.839 | 0.00% |
| Polson et al. (2018) | 0.047 [-0.032,0.126] | 0.245 | 45.40% |
| **Pooled estimate** | **0.054 [-0.018,0.126]** | **0.143** | **32.00%** |
| **Race** |  |  |  |
| Bartley et al 2019 | 0.108 [-0.015,0.228] | 0.086 | 0.00% |
| Fullen et al 2018 | -0.046 [-0.271,0.184] | 0.698 | 34.90% |
| Polson et al 2018 | 0 [-0.263,0.264] | 0.998 | 70.90% |
| **Pooled estimate** | **0.034 [-0.129,0.195]** | **0.682** | **42.20%** |
| **Education** |  |  |  |
| Bartley et al 2019 | 0.085 [0.032,0.137] | 0.002 | 48.60% |
| Fullen et al 2018 | 0.083 [0.023,0.142] | 0.007 | 52.90% |
| Lamond et al 2008 | 0.113 [0.068,0.159] | < 0.001 | 31.90% |
| Li et al 2015 | 0.102 [0.05,0.153] | < 0.001 | 34.20% |
| Silverman et al 2015 | 0.066 [0.019,0.111] | 0.005 | 32.70% |
| **Pooled estimate** | **0.09 [0.039,0.141]** | **< 0.001** | **43.00%** |
| **Marital status** |  |  |  |
| Bartley et al. (2019) | 0.098 [0.002,0.192] | 0.0445 | 67.90% |
| King & Richardson (2016) | -0.143 [-0.525,0.288] | 0.5214 | 91.30% |
| Liddell & Ferreira (2019) | -0.099 [-0.565,0.415] | 0.72 | 93.20% |
| **Pooled estimate** | **-0.033 [-0.329,0.269]** | **0.8354** | **86.50%** |
| **Income** |  |  |  |
| Bartley et al. (2019) | 0.157 [0.098,0.215] | < 0.001 | 24.60% |
| King & Richardson (2016) | 0.28 [-0.045,0.552] | 0.09023 | 84.60% |
| Silverman et al. (2015) | 0.311 [0.06,0.526] | 0.01601 | 72.10% |
| **Pooled estimate** | **0.234 [0.075,0.382]** | **0.00416** | **73.10%** |
| **Psychological distress** |  |  |  |
| Baldwin et al. (2011) | -0.462 [-0.603,-0.292] | <0.001 | 59.70% |
| Moore et al. (2015) | -0.38 [-0.512,-0.231] | <0.001 | 0.00% |
| Ong et al. (2006) (Study 1a) | -0.471 [-0.599,-0.32] | <0.001 | 60.70% |
| Ong et al. (2006) (Study 1b) | -0.496 [-0.61,-0.362] | <0.001 | 43.00% |
| Ong et al. (2006) (Study 2) | -0.473 [-0.603,-0.32] | <0.001 | 58.60% |
| **Pooled estimate** | **-0.463 [-0.582,-0.326]** | **<0.001** | **55.20%** |
| **Optimism** |  |  |  |
| Baldwin et al. (2011) | 0.573 [0.325,0.747] | <0.001 | 97.70% |
| Lamond et al. (2008) | 0.585 [0.338,0.757] | <0.001 | 94.20% |
| Lee et al. (2008) | 0.572 [0.307,0.754] | <0.001 | 97.70% |
| Lu et al. (2017) | 0.443 [0.403,0.481] | <0.001 | 0.00% |
| **Pooled estimate** | **0.549 [0.354,0.698]** | **<0.001** | **96.60%** |
| **Spirituality** |  |  |  |
| Lee et al. (2008) | 0.279 [0.238,0.319] | < 0.001 | 0.00% |
| Vahia et al. (2011) | 0.205 [0.083,0.32] | 0.001 | 0.00% |
| Polson et al. (2018) | 0.276 [0.236,0.315] | < 0.001 | 0.00% |
| **Pooled estimate** | **0.269 [0.22,0.317]** | **< 0.001** | **0.00%** |
| **Positive daily emotions** |  |  |  |
| Ong et al. (2006) (Study 1a) | 0.388 [0.171,0.57] | < 0.001 | 0.00% |
| Ong et al. (2006) (Study 1b) | 0.41 [0.17,0.604] | 0.001 | 0.00% |
| Ong et al. (2006) (Study 2) | 0.386 [0.155,0.577] | 0.001 | 0.00% |
| **Pooled estimate** | **0.394 [0.209,0.552]** | **< 0.001** | **0.00%** |
| **Negative daily emotions** |  |  |  |
| Ong et al. (2006) (Study 1a) | -0.209 [-0.438,0.046] | 0.107 | 14.80% |
| Ong et al. (2006) (Study 1b) | -0.243 [-0.472,0.017] | 0.066 | 0.00% |
| Ong et al. (2006) (Study 2) | -0.098 [-0.336,0.152] | 0.443 | 0.00% |
| **Pooled estimate** | **-0.182 [-0.37,0.02]** | **0.077** | **0.00%** |
| **Morale** |  |  |  |
| Wagnild (2003) (low income) | 0.275 [0.168,0.376] | <0.001 | 57.80% |
| Wagnild (2003) (high income) | 0.323 [0.231,0.41] | <0.001 | 22.40% |
| Wagnild (2003) (low income) | 0.26 [0.159,0.356] | <0.001 | 46.30% |
| Wagnild & Young (1993) | 0.308 [0.131,0.465] | <0.001 | 66.20% |
| **Pooled estimate** | **0.291 [0.191,0.384]** | **<0.001** | **49.40%** |
| **Health promoting lifestyle** |  |  |  |
| Wagnild (2003) (high income) | 0.528 [0.394,0.641] | <0.001 | 0.00% |
| Wagnild (2003) (low income) | 0.529 [0.434,0.612] | <0.001 | 0.00% |
| Wagnild & Torma (2013) | 0.53 [0.449,0.602] | <0.001 | 0.00% |
| **Pooled estimate** | **0.529 [0.451,0.6]** | **<0.001** | **0.00%** |

**Supplementary Figure 10a.** Publication bias analysis: age

**Age**


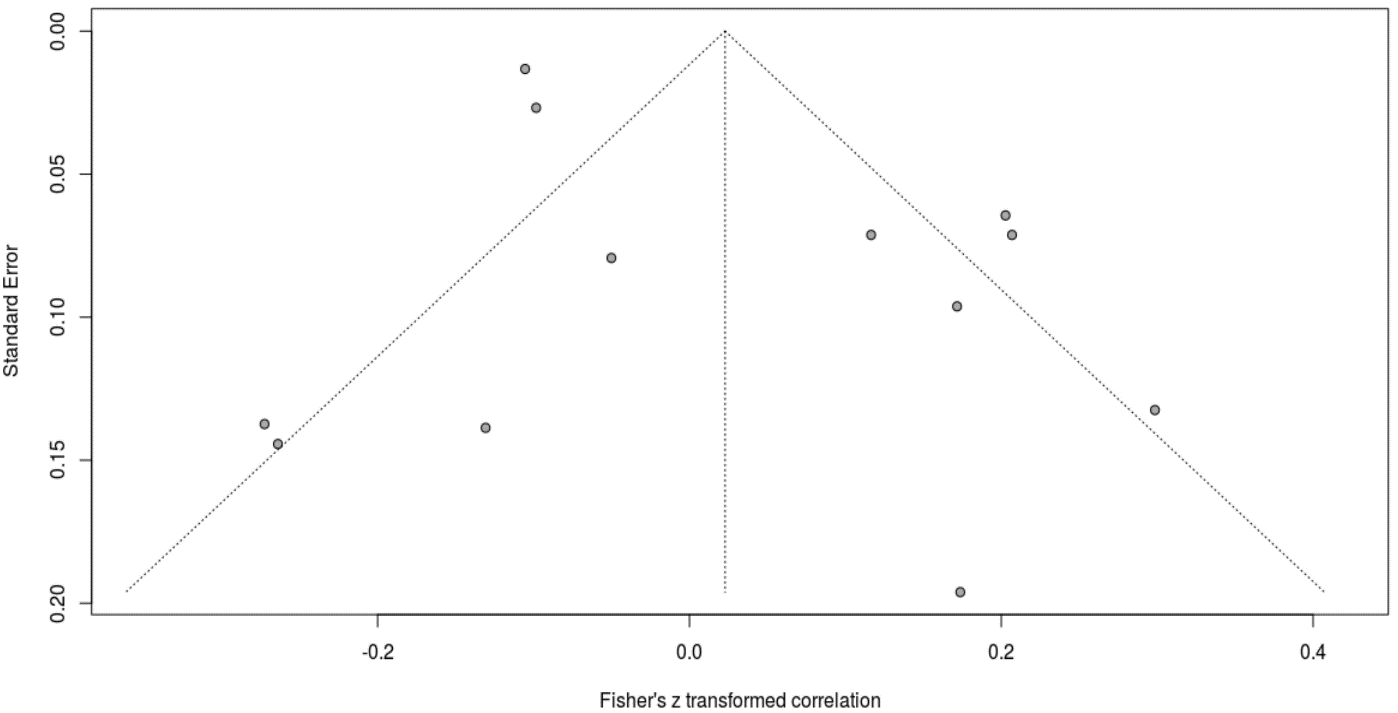


**Supplementary Figure 10b.** Publication bias analysis: depressive symptoms

**Depressive symptoms**


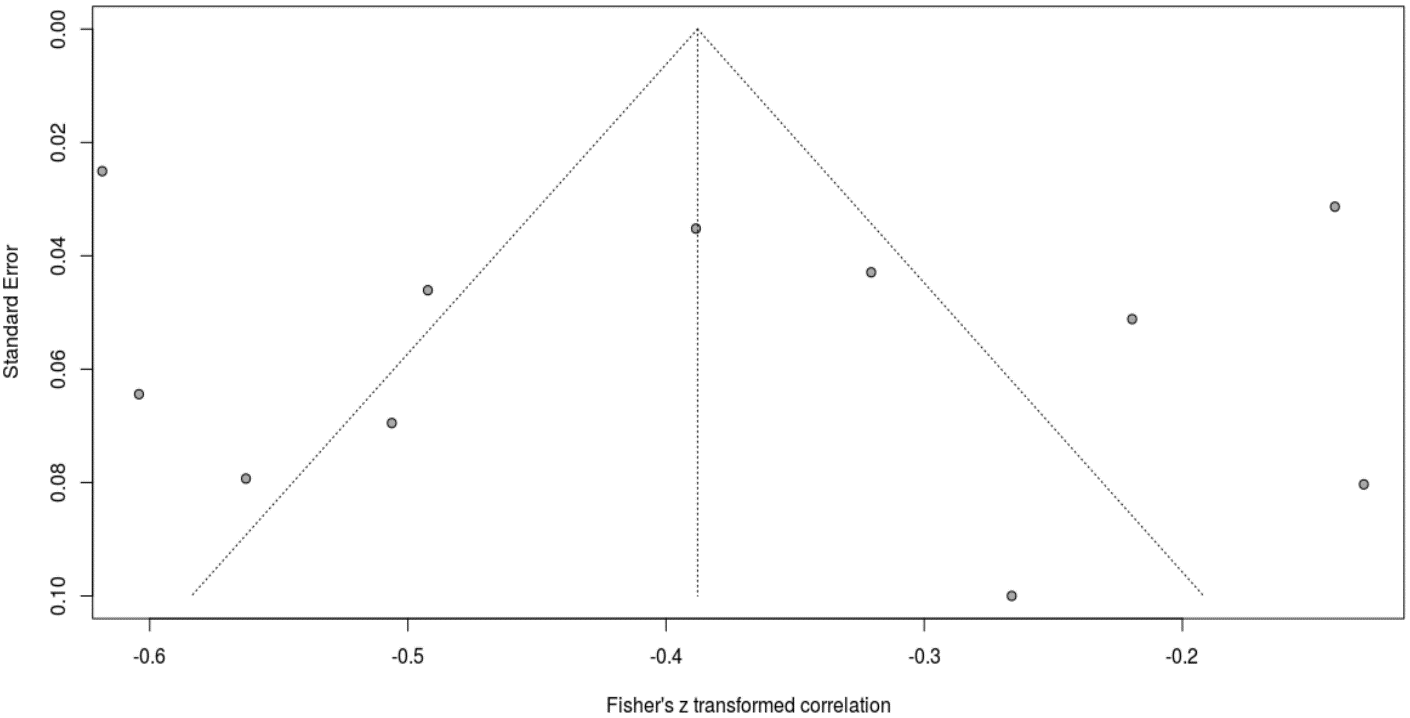


**Supplementary Figure 10c.** Publication bias analysis: self-rated health total

**Self-rated health total**


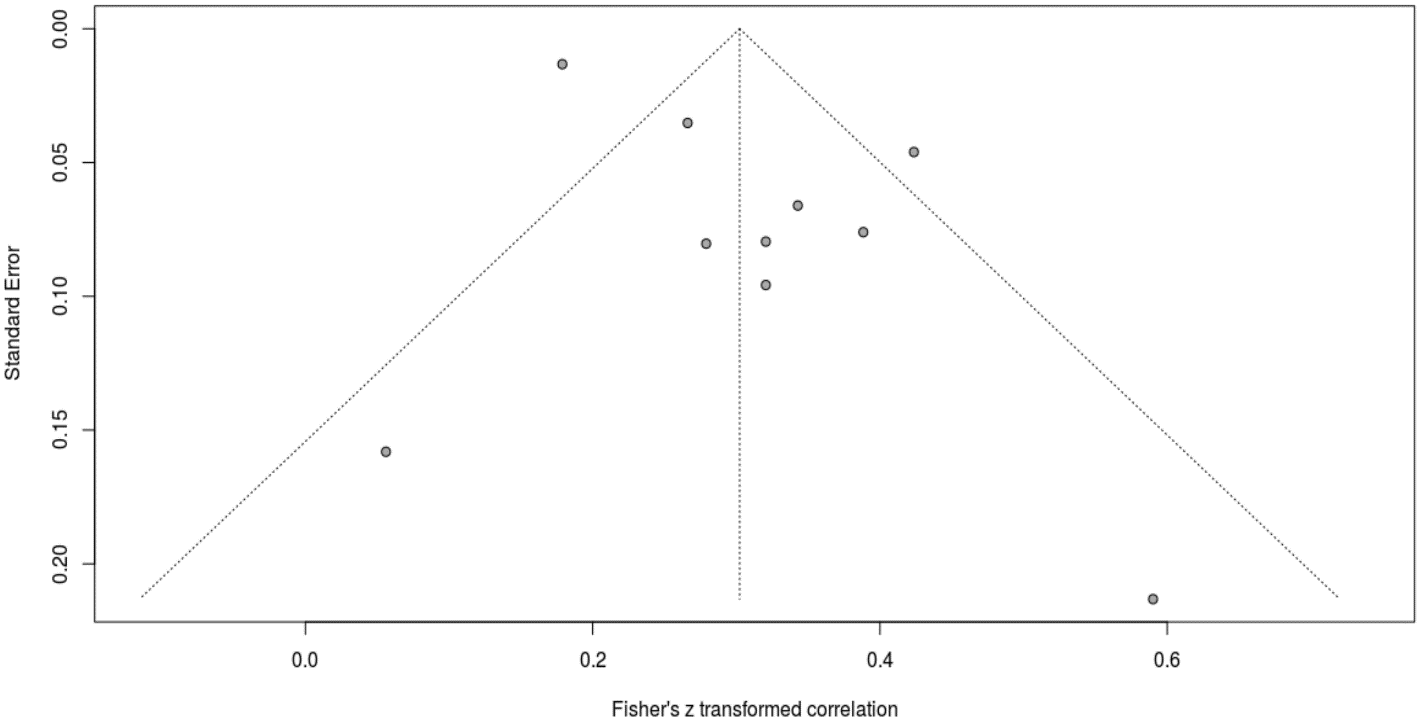


**Supplementary Figure 10d.** Publication bias analysis: self-rated physical health

**Self-rated physical health**


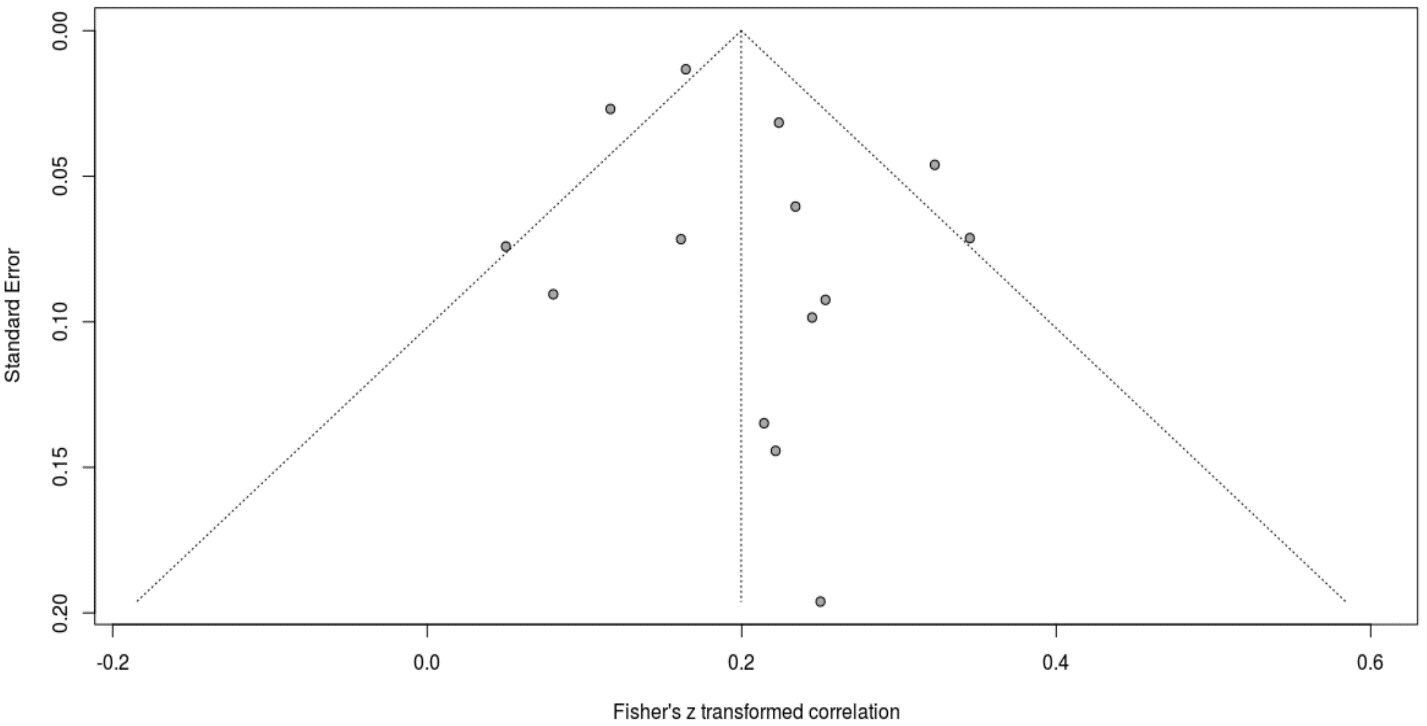


**Supplementary Figure 10e.** Publication bias analysis: self-rated mental health

**Self-rated mental health**

**
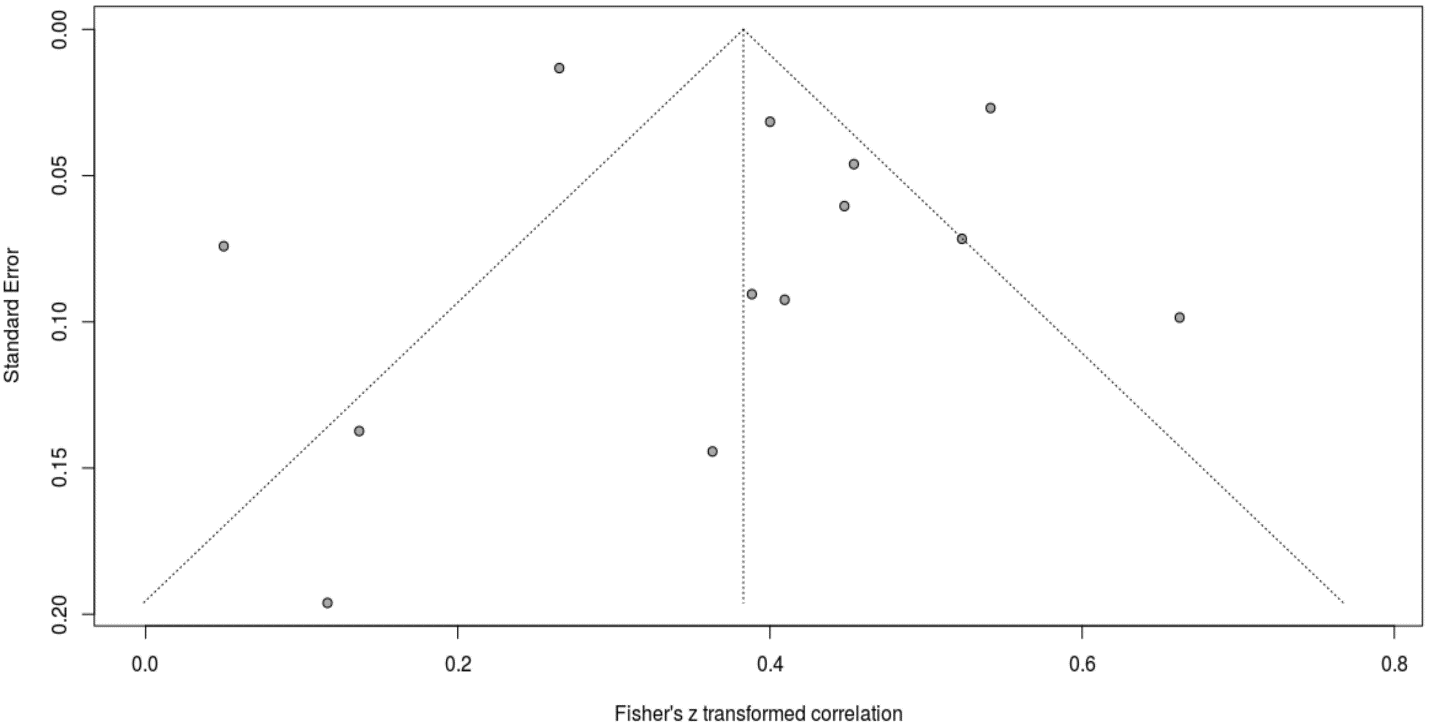
**

**Supplementary References**

Aromataris, E, & Munn, Z. (2020). *JBI Manual for Evidence Synthesis*. https://synthesismanual.jbi.global. https://doi.org/10.46658/JBIMES-20-01

Baldwin, D. R., Jackson III, D., Okoh, I., & Cannon, R. L. (2011). Resiliency and optimism: An African American senior citizen’s perspective. *Journal of Black Psychology*, *37*(1), 24-41.

Bane, M. S. (1998). *Relationship between psychological resilience and social support in predicting affect and health in older adults*. Dissertation.

Bartley, E. J., Palit, S., Fillingim, R. B., & Robinson, M. E. (2019). Multisystem Resiliency as a Predictor of Physical and Psychological Functioning in Older Adults with Chronic Low Back Pain. *Frontiers in psychology*, *10*, 1932.

Bartone, P. T. (1991, June). *Development and validation of a short hardiness measure*. In annual convention of the American Psychological Society. Washington DC.

Bartone, l. T. (1995, June). *A short hardiness scale*. Paper presented at the American Psychological Society Annual Convention, New York.

Bartone, P. T. (2007). Test-retest reliability of the dispositional resilience scale-15, a brief hardiness scale. *Psychological reports*, *101*(3), 943-944.

Bartone, P. T., Ursano, R. J., Wright, K. M., & Ingraham, L. H. (1989). The impact of a military air disaster on the health of assistance workers. *Journal of nervous and mental disease*, *177*(6), 317-328.

Bernstein, K., Park, S. Y., & Nokes, K. M. (2017). Resilience and depressive symptoms among Korean Americans with history of traumatic life experience. *Community mental health journal*, *53*(7), 793-801.

Beutel, M. E., Glaesmer, H., Decker, O., Fischbeck, S., & Brähler, E. (2009). Life satisfaction, distress, and resiliency across the life span of women. *Menopause*, *16*(6), 1132-1138.

Block, J. (2002). *Personality as an affect-processing system: Toward an integrative theory*. New York: Psychology Press.

Block, J., & Kremen, A. M. (1996). IQ and ego-resiliency: conceptual and empirical connections and separateness. *Journal of personality and social psychology*, *70*(2), 349.

Boseman, G. (2001). *Correlates of morale in elderly African-American women*. Dissertation.

Brennan, S. L., Irwin, J., Drincic, A., Amoura, N. J., Randall, A., & Smith-Sallans, M. (2017). Relationship among gender-related stress, resilience factors, and mental health in a Midwestern US transgender and gender-nonconforming population. *International Journal of Transgenderism*, *18*(4), 433-445.

Campbell-Sills, L., Forde, D. R., & Stein, M. B. (2009). Demographic and childhood environmental predictors of resilience in a community sample. *Journal of psychiatric research*, *43*(12), 1007-1012.

Campbell‐Sills, L., & Stein, M. B. (2007). Psychometric analysis and refinement of the Connor–Davidson resilience scale (CD‐RISC): Validation of a 10‐item measure of resilience. *Journal of Traumatic Stress: Official Publication of the International Society for Traumatic Stress Studies*, *20*(6), 1019-1028.

Carandang, R. R., Shibanuma, A., Kiriya, J., Asis, E., Chavez, D. C., Meana, M., ... & Jimba, M. (2019). Determinants of depressive symptoms in Filipino senior citizens of the community-based ENGAGE study. *Archives of gerontology and geriatrics*, *82*, 186-191.

Clark, P. G., Greene, G. W., Blissmer, B. J., Lees, F. D., Riebe, D. A., & Stamm, K. E. (2019). Trajectories of maintenance and resilience in healthful eating and exercise behaviors in older adults. *Journal of aging and health*, *31*(5), 861-882.

Connor, K. M., & Davidson, J. R. (2003). Development of a new resilience scale: The Connor‐Davidson resilience scale (CD‐RISC). *Depression and anxiety*, *18*(2), 76-82.

Cunha, G. L. D., Cabral, S. M., & Santos, G. A. D. (2017). Analysis of the relationship between the syndrome of frailty and cognitive deficit in older adults with independent living. *Análise Psicológica*, *35*(1), 37-43.

Davidson, J.R. (2020, January 1). *Connor-Davidson Resilience Scale (CD-RISC) Manual*. Retrieved from: www.cd-risc.com.

Driver, S., Warren, A. M., Reynolds, M., Agtarap, S., Hamilton, R., Trost, Z., & Monden, K. (2016). Identifying predictors of resilience at inpatient and 3-month post-spinal cord injury. *The journal of spinal cord medicine*, *39*(1), 77-84.

Ebly, E. M., Hogan, D. B., & Fung, T. S. (1996). Correlates of self-rated health in persons aged 85 and over: results from the Canadian Study of Health and Aging. *Canadian journal of public health = Revue canadienne de sante publique*, *87*(1), 28-31.

Emlet, C. A., Shiu, C., Kim, H. J., & Fredriksen-Goldsen, K. (2017). Bouncing back: resilience and mastery among HIV-positive older gay and bisexual men. *The Gerontologist,* *57*(suppl_1), S40-S49.

Eshel, Y., Kimhi, S., Lahad, M., Leykin, D., & Goroshit, M. (2018). Risk factors as major determinants of resilience: A replication study. *Community mental health journal*, *54*(8), 1228-1238.

Freitag, S., & Schmidt, S. (2016). Psychosocial correlates of frailty in older adults. *Geriatrics*, *1*(4), 26.

Fullen, M. C. (2016). “*Gray Hair is a Crown of Glory”: A Multivariate Analysis of Wellness, Resilience, and Internalized Ageism in Older Adulthood* (Doctoral dissertation, The Ohio State University).

Fullen, M. C., & Granello, D. H. (2018). Holistic wellness in older adulthood: group differences based on age and mental health. *Journal of Holistic Nursing*, *36*(4), 395-407.

Fullen, M.C., Richardson, V.E. & Granello, D.H. (2018). Comparing successful aging, resilience, and holistic wellness as predictors of the good life. *Educational Gerontology*, *44*(7), 459-468.

Gooding, P. A., Hurst, A., Johnson, J., & Tarrier, N. (2012). Psychological resilience in young and older adults. *International journal of geriatric psychiatry*, *27*(3), 262-270.

Guest, R., Craig, A., Tran, Y., & Middleton, J. (2015). Factors predicting resilience in people with spinal cord injury during transition from inpatient rehabilitation to the community. Spinal Cord, *53*(9), 682-686.

Hardy, S. E., Concato, J., & Gill, T. M. (2002). Stressful life events among community‐living older persons. *Journal of General Internal Medicine*, *17*(11), 841-847.

Hardy, S. E., Concato, J., & Gill, T. M. (2004). Resilience of community‐dwelling older persons. *Journal of the American Geriatrics Society*, *52*(2), 257-262.

Heisel, M. J., Moore, S. L., Flett, G. L., Norman, R. M. G., Links, P. S., Eynan, R., ... & Farrell, B. (2020). Meaning-centered men’s groups: Initial findings of an intervention to enhance resiliency and reduce suicide risk in men facing retirement. *Clinical gerontologist*, *43*(1), 76-94.

Holden, K. B., Bradford, L. D., Hall, S. P., & Belton, A. S. (2013). Prevalence and correlates of depressive symptoms and resiliency among African American women in a community-based primary health care center. *Journal of health care for the poor and underserved*, *24*(4 0), 79.

Holmes, S. D., Galik, E., & Resnick, B. (2019). The mediating effect of resilience between social support for exercise and resident satisfaction in assisted living. *Journal of Housing For the Elderly*, *33*(1), 56-71.

Jeste, D. V., Savla, G. N., Thompson, W. K., Vahia, I. V., Glorioso, D. K., Martin, A. V. S., ... & Depp, C. A. (2013). Association between older age and more successful aging: critical role of resilience and depression. *American Journal of Psychiatry*, *170*(2), 188-196.

Jeste, D.V., Glorioso, D., Lee, E.E., Daly, R., Graham, S., Liu, J., Paredes, A.M., Nebeker, C., Tu, X.M., Twamley, E.W. & Van Patten, R. (2019). Study of independent living residents of a continuing care senior housing community: Sociodemographic and clinical associations of cognitive, physical, and mental health. *The American Journal of Geriatric Psychiatry*, *27*(9), 895-907.

Johnson, J. D. (2002). *Relationship between resiliency, self-care agency, and self-care in the community-dwelling older adult*. Dissertation.

Johnson, J., Gooding, P., & Tarrier, N. (2008). Suicide risk in schizophrenia: explanatory models and clinical implications, The Schematic Appraisal Model of Suicide (SAMS). *Psychology and Psychotherapy: Theory, Research and Practice*, *81*(1), 55-77.

Johnson, J., Gooding, P. A., Wood, A. M., & Tarrier, N. (2010). Resilience as positive coping appraisals: Testing the schematic appraisals model of suicide (SAMS). *Behaviour Research and Therapy*, *48*(3), 179-186.

Kilic, S. A., Dorstyn, D. S., & Guiver, N. G. (2013). Examining factors that contribute to the process of resilience following spinal cord injury. *Spinal cord*, *51*(7), 553-557.

Klinedinst, N. J., & Resnick, B. (2014). Volunteering and depressive symptoms among residents in a continuing care retirement community*. Journal of gerontological social work*, *57*(1), 52-71.

King, S.D., & Richardson, V.E. (2016). Influence of income, being partnered/married, resilience, and discrimination on mental health distress for midlife and older gay men: Mental health distress among midlife and older gay men: The importance of partners and resilience. *Journal of Gay & Lesbian Mental Health*, *20*(2), 127-151.

Kuwert, P., Knaevelsrud, C., & Pietrzak, R.H. (2014). Loneliness among older veterans in the United States: results from the National Health and Resilience in Veterans Study. *The American Journal of Geriatric Psychiatry*, *22*(6), 564-569.

Lamet, A., Szuchman, L., Perkel, L., & Walsh, S. (2008). Risk factors, resilience, and psychological distress among holocaust and nonholocaust surviviors in the post-9/11 environment. *Educational Gerontology*, *35*(1), 32-46.

Lamond, A.J., Depp, C.A., Allison, M., Langer, R., Reichstadt, J., Moore, D.J., Golshan, S., Ganiats, T.G., & Jeste, D.V. (2008). Measurement and predictors of resilience among community-dwelling older women. *Journal of psychiatric research*, *43*(2), 148-154.

Lau, R., Morse, C. A., & Macfarlane, S. (2010). Psychological factors among elderly women with suicidal intentions or attempts to suicide: a controlled comparison. *Journal of women & aging*, *22*(1), 3-14.

Lau, S. Y. Z., Guerra, R. O., de Souza Barbosa, J. F., & Phillips, S. P. (2018). Impact of resilience on health in older adults: a cross-sectional analysis from the International Mobility in Aging Study (IMIAS). *BMJ open*, *8*(11), e023779.

Lee, H.S., Brown, S.L., Mitchell, M.M., & Schiraldi, G.R. (2008). Correlates of resilience in the face of adversity for Korean women immigrating to the US. *Journal of Immigrant and Minority Health*, *10*(5), 415-422.

Lee M.E., Jung M.H., Kim S., & Kim B. (2018). Risk and protective factors affecting suicide attempts among Korean elderly living alone in urban areas. *Asia life sciences*, *27*(2), 249-261.

Lei P. Q., Liu C.-N., Gao Y., & Xue M. (2018). Psychosocial Factors and Frailty in Community-dwelling Older People. *Chinese General Practice*, *21*(2), 180.

Li, J., Theng, Y.L., & Foo, S. (2015). Depression and psychosocial risk factors among community-dwelling older adults in Singapore. *Journal of cross-cultural gerontology*, *30*(4), 409-422.

Liddell, J., & Ferreira, R.J. (2019). Predictors of Individual resilience characteristics among individuals ages 65 and older in post-disaster settings. *Disaster medicine and public health preparedness*, *13*(2), 256-264.

Liebenberg, L., & Moore, J. C. (2018). A social ecological measure of resilience for adults: The RRC-ARM. *Social Indicators Research*, *136*(1), 1-19.

Lim, M.L., Lim, D., Gwee, X., Nyunt, M.S.Z., Kumar, R., & Ng, T.P. (2015). Resilience, stressful life events, and depressive symptomatology among older Chinese adults. *Aging & mental health*, *19*(11), 1005-1014.

Lu, C., Yuan, L., Lin, W., Zhou, Y., & Pan, S. (2017). Depression and resilience mediates the effect of family function on quality of life of the elderly. *Archives of gerontology and geriatrics*, *71*, 34-42.

Manning, L.K., Carr, D.C., & Kail, B.L. (2016). Do higher levels of resilience buffer the deleterious impact of chronic illness on disability in later life? *The Gerontologist*, *56*(3), 514-524.

Marciano, H., Kimhi, S., & Eshel, Y. (2019). Predictors of individual, community and national resiliencies of Israeli Jews and Arabs. *International Journal of Psychology.* *55*(4), 553–561.

Martin, A.S., Palmer, B.W., Rock, D., Gelston, C.V., & Jeste, D.V. (2015b). Associations of self-perceived successful aging in young-old versus old-old adults. *International psychogeriatrics*, *27*(4), 601-609.

Martins, A. B., Dos Santos, C. M., Hilgert, J. B., de Marchi, R. J., Hugo, F. N., & Pereira Padilha, D. M. (2011). Resilience and self‐perceived oral health: a hierarchical approach. *Journal of the American Geriatrics Society*, *59*(4), 725-731.

McClain, J., Gullatt, K., & Lee, C. (2018). *Resilience and Protective Factors in Older Adults*. [master thesis] USA: University of California.

McKibbin, C., Lee, A., Steinman, B.A., Carrico, C., Bourassa, K., & Slosser, A. (2016). Health status and social networks as predictors of resilience in older adults residing in rural and remote environments. *Journal of aging research*, *2016*, 4305894.

Mehta, M., Whyte, E., Lenze, E., Hardy, S., Roumani, Y., Subashan, P., Huang, W., & Studenski, S. (2008). Depressive symptoms in late life: associations with apathy, resilience and disability vary between young‐old and old‐old. *International Journal of Geriatric Psychiatry: A journal of the psychiatry of late life and allied sciences*, *23*(3), 238-243.

Mertens, V. C., Bosma, H., Groffen, D. A., & van Eijk, J. T. M. (2012). Good friends, high income or resilience? What matters most for elderly patients?.*The European Journal of Public Health*, *22*(5), 666-671.

Min, J. H., Ha, L. M., & Young, K. H. (2017). Factors Influencing Self-care in Elders with Hypertension Living at Home. *Journal of Korean Academy of Fundamentals of Nursing*, *24*(1), 72-83.

Moe, A., Hellzen, O., Ekker, K., & Enmarker, I. (2013). Inner strength in relation to perceived physical and mental health among the oldest old people with chronic illness. *Aging & Mental Health*, *17*(2), 189-196.

Moher D, Liberati A, Tetzlaff J, Altman DG, The PRISMA Group (2009). Preferred Reporting Items for Systematic Reviews and Meta-Analyses: The PRISMA Statement. *PLoS Med,* *6*(7): e1000097.

Montross, L.P., Depp, C., Daly, J., Reichstadt, J., Golshan, S., Moore, D., Sitzer, D., & Jeste, D.V. (2006). Correlates of self-rated successful aging among community-dwelling older adults. *The American Journal of Geriatric Psychiatry*, *14*(1), 43-51.

Moore, R.C., Eyler, L.T., Mausbach, B.T., Zlatar, Z.Z., Thompson, W.K., Peavy, G., Fazeli, P.L., & Jeste, D.V. (2015). Complex interplay between health and successful aging: role of perceived stress, resilience, and social support. *The American Journal of Geriatric Psychiatry*, *23*(6), 622-632.

Morote, R., Hjemdal, O., Uribe, P. M., & Corveleyn, J. (2017). Psychometric properties of the Resilience Scale for Adults (RSA) and its relationship with life-stress, anxiety and depression in a Hispanic Latin-American community sample. *PloS one*, *12*(11).

NIH. (2020). *Study Quality Assessment Tools: Quality Assessment Tool for Observational Cohort and Cross-Sectional Studies*. https://www.nhlbi.nih.gov/health-topics/study-quality-assessment-tools

Netuveli, G., Wiggins, R. D., Montgomery, S. M., Hildon, Z., & Blane, D. (2008). Mental health and resilience at older ages: Bouncing back after adversity in the British Household Panel Survey. *Journal of Epidemiology & Community Health*, *62*(11), 987-991.

Nygren, B., Aléx, L., Jonsén, E., Gustafson, Y., Norberg, A., & Lundman, B. (2005). Resilience, sense of coherence, purpose in life and self-transcendence in relation to perceived physical and mental health among the oldest old. *Aging & mental health*, *9*(4), 354-362.

Ong, A.D., Bergeman, C.S., Bisconti, T.L., & Wallace, K.A. (2006). Psychological resilience, positive emotions, and successful adaptation to stress in later life. *Journal of personality and social psychology*, *91*(4), 730.

Pakenham, K. I., Mawdsley, M., Brown, F. L., & Burton, N. W. (2018). Pilot evaluation of a resilience training program for people with multiple sclerosis. *Rehabilitation psychology*, *63*(1), 29.

de Paula Couto, M. C. P., Koller, S. H., & Novo, R. (2011). Stressful life events and psychological well-being in a Brazilian sample of older persons: The role of resilience. *Aging international*, *36*(4), 492-505.

Pearlin, L.I., & Schooler, C. (1978). The structure of coping. *Journal of Health and Social Behavior*, *19*, 2-21.

Phillips, S. P., Auais, M., Belanger, E., Alvarado, B., & Zunzunegui, M. V. (2016). Life-course social and economic circumstances, gender, and resilience in older adults: The longitudinal International Mobility in Aging Study (IMIAS). *SSM-Population Health*, *2*, 708-717.

Pietrzak, R. H., Tsai, J., Kirwin, P. D., & Southwick, S. M. (2014). Successful aging among older veterans in the United States. *The American Journal of Geriatric Psychiatry*, *22*(6), 551-563.

Polson, E.C., Gillespie, R., & Myers, D.R. (2018). Hope and Resilience among Vulnerable, Community-Dwelling Older Persons. *Social Work & Christianity*, *45*(1).

Prince-Embury, S. (2013). The ego-resiliency scale by Block and Kremen (1996) and trait ego-resiliency. In S. Prince-Embury & D.H. Saklofske (Eds.), *Resilience in children, adolescents, and adults* (pp. 135-138). New York: Springer.

Resnick, B. A., & Inguito, P. L. (2011). The Resilience Scale: Psychometric properties and clinical applicability in older adults. *Archives of psychiatric nursing*, *25*(1), 11-20.

Reyes, A. T., Constantino, R. E., Cross, C. L., Tan, R. A., Bombard, J. N., & Acupan, A. R. (2019). Resilience and psychological trauma among Filipino American women. *Archives of psychiatric nursing*, *33*(6), 177-185.

Roos, N. P., & Havens, B. (1991). Predictors of successful aging: a twelve-year study of Manitoba elderly. *American Journal of Public Health*, *81*(1), 63-68.

Rossi, N.E., Bisconti, T.L., & Bergeman, C.S. (2007). The role of dispositional resilience in regaining life satisfaction after the loss of a spouse. *Death studies*, *31*(10), 863-883.

Rowe, J. W., & Kahn, R. L. (1997). Successful aging. *The Gerontologist*, *37*(4), 433-440.

Sawyer, P., & Allman, R. M. (2010). 12 Resilience in mobility in the context of chronic disease and aging: cross-sectional and prospective findings from the University of Alabama at Birmingham (UAB) Study of Aging. *New Frontiers in Resilient Aging: Life-Strengths and Well-Being in Late Life*, *310*.

Scelzo, A., Di Somma, S., Antonini, P., Montross, L.P., Schork, N., Brenner, D., & Jeste, D.V. (2018). Mixed-methods quantitative–qualitative study of 29 nonagenarians and centenarians in rural Southern Italy: focus on positive psychological traits. *International Psychogeriatrics*, *30*(1), 31-38.

Schure, M.B., Odden, M., & Goins, R.T. (2013). The association of resilience with mental and physical health among older American Indians: The native elder care study. *American Indian and Alaska native mental health research*, *20*(2), 27.

Serrano-Parra, M. D., Garrido-Abejar, M., Notario-Pacheco, B., Bartolomé-Gutierrez, R., Solera-Martínez, M., & Martínez-Vizcaino, V. (2012). Validez de la escala de Resiliencia de Connor-Davidson (CD-RISC) en una población de mayores entre 60 y 75 años. *International Journal of psychological research*, *5*(2), 49-57.

Sharpley, C. F., Palanisamy, S. K., Metcalf, K., Jones, K. A., Kelly, B., & McFarlane, J. R. (2014). A comparison of a single genetic factor, two stress factors, and one psychosocial coping factor as predictors of depression in an Australian community sample. *Archives of Psychiatry and Psychotherapy*, *4*(16), 15-26.

Shen, K., & Zeng, Y. (2011). The association between resilience and survival among Chinese elderly. In *Resilience in Aging* (pp. 217-229). Springer, New York, NY.

Silverman, A.M., Molton, I.R., Alschuler, K.N., Ehde, D.M., & Jensen, M.P. (2015). Resilience predicts functional outcomes in people aging with disability: A longitudinal investigation. *Archives of physical medicine and rehabilitation*, *96*(7), 1262-1268.

Smith, P.R. (2009). Resilience: resistance factor for depressive symptom. *Journal of Psychiatric and Mental Health Nursing*, *16*(9), 829-837.

Smith, P.R. (2012). Resilience and stigma influence older African Americans seeking care. *ABNF Journal,* *23*(4).

Smith, B. W., Dalen, J., Wiggins, K., Tooley, E., Christopher, P., & Bernard, J. (2008). The brief resilience scale: assessing the ability to bounce back. *International journal of behavioral medicine*, *15*(3), 194-200.

Smith, J. L., & Hollinger-Smith, L. (2015). Savoring, resilience, and psychological well-being in older adults. *Aging & mental health*, *19*(3), 192-200.

Spies, G., & Seedat, S. (2014). Depression and resilience in women with HIV and early life stress: Does trauma play a mediating role? A cross-sectional study. *BMJ open*, *4*(2), e004200.

Stewart, J. M., Auais, M., Belanger, E., & Phillips, S. P. (2019). Comparison of self-rated and objective successful aging in an international cohort. *Aging & Society*, *39*(7), 1317-1334.

Sun, J., & Buys, N. (2014). A comparison between a Tai Chi program and a usual medical care program in chronic cardiovascular disease participants in quality of life, psychological health, resilience, blood pressure and body mass index. *International Journal on Disability and Human Development*, *13*(1), 113-120.

Talsma, A. N. (1996). *Evaluation of a theoretical model of resilience and select predictors of resilience in a sample of community-based elderly*. Doctoral dissertation: The University of Michigan.

Tomás, J. M., Sancho, P., Melendez, J. C., & Mayordomo, T. (2012). Resilience and coping as predictors of general well-being in the elderly: A structural equation modeling approach. *Aging & Mental Health*, *16*(3), 317-326.

Topel, M. L., Kim, J. H., Mujahid, M. S., Ko, Y. A., Vaccarino, V., Mubasher, M., ... & Quyyumi, A. A. (2019). Individual Characteristics of Resilience are Associated With Lower‐Than‐Expected Neighborhood Rates of Cardiovascular Disease in Blacks: Results From the Morehouse‐Emory Cardiovascular (MECA) Center for Health Equity Study. *Journal of the American Heart Association*, *8*(12), e011633.

Torma, L.M., Houck, G.M., Wagnild, G.M., Messecar, D., & Jones, K.D. (2013). Growing old with fibromyalgia: factors that predict physical function. *Nursing research*, *62*(1), 16-24.

Tugade, M. M., Fredrickson, B. L., & Feldman Barrett, L. (2004). Psychological resilience and positive emotional granularity: Examining the benefits of positive emotions on coping and health. *Journal of personality*, *72*(6), 1161-1190.

Vahia, I.V., Depp, C.A., Palmer, B.W., Fellows, I., Golshan, S., Thompson, W., Allison, M., & Jeste, D.V. (2011). Correlates of spirituality in older women. *Aging & mental health*, *15*(1), 97-102.

Vahia, I. V., Meeks, T. W., Thompson, W. K., Depp, C. A., Zisook, S., Allison, M., ... & Jeste, D. V. (2010). Subthreshold depression and successful aging in older women. *The American Journal of Geriatric Psychiatry*, *18*(3), 212-220.

Wagnild, G. (2003). Resilience and successful aging: Comparison among low and high income older adults. *Journal of gerontological nursing*, *29*(12), 42-49.

Wagnild, G.M., & Torma, L.M. (2013). Assessing resilience in older frontier women. In A.C Winters & H.J. Lee (Eds.), *Rural nursing: Concepts, theory, and practice* (pp. 79-94).

Wagnild, G. M., & Young, H. M. (1993). Development and psychometric evaluation of the Resilience Scale. *Journal of Nursing Measurement*, *1*(2), 165-178.

Wallace, K. A. (1999). *A theoretical examination of protective factors in later life*. Doctoral dissertation: University of Notre Dame.

Wallace, K.A., Bisconti, T.L., & Bergeman, C.S. (2001). The mediational effect of hardiness on social support and optimal outcomes in later life. *Basic and applied social psychology*, *23*(4), 267-276.

Wells, M. (2009). Resilience in rural community‐dwelling older adults. *The Journal of Rural Health*, *25*(4), 415-419.

Wells, M. (2010). Resilience in older adults living in rural, suburban, and urban areas. *Online Journal of Rural Nursing and Health Care*, *10*(2), 45-54.

Wisco, B. E., Marx, B. P., Wolf, E. J., Miller, M. W., Southwick, S. M., & Pietrzak, R. H. (2014). Posttraumatic stress disorder in the US veteran population: results from the National Health and Resilience in Veterans Study. *The Journal of clinical psychiatry*, *75*(12), 1338-1346.

Yang, Y., & Wen, M. (2015). Psychological resilience and the onset of activity of daily living disability among older adults in China: A nationwide longitudinal analysis. *Journals of Gerontology Series B: Psychological Sciences and Social Sciences*, *70*(3), 470-480.

You, S., & Park, M. (2017). Resilience protected against suicidal behavior for men but not women in a community sample of older adults in Korea. *Frontiers in psychology*, *8*, 401.

Zeng, Y., & Shen, K. (2010). Resilience significantly contributes to exceptional longevity. *Current gerontology and geriatrics research*, *2010*, 525693.
